# Supplementary material for: sn-spMF: matrix factorization informs tissue-specific genetic regulation of gene expression
Source: Genome Biol. 2020 Sep 11;21:235. doi: 10.1186/s13059-020-02129-6 (PMC7488540; doi:10.1186/s13059-020-02129-6)
Supplement: Supplementary file 1 — Additional file 1 Supplementary figure. Figure S1 - S38. [file 13059_2020_2129_MOESM1_ESM.pdf]

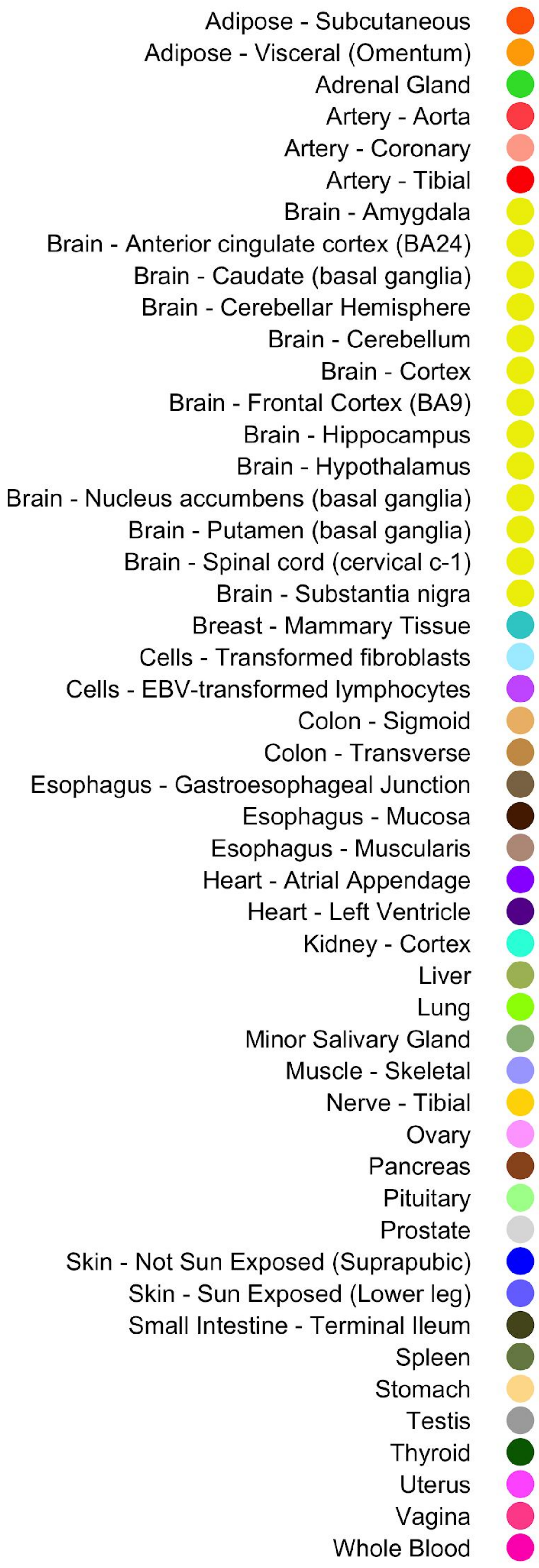

Figure S1. Color scheme of the 49 tissues in GTEx.

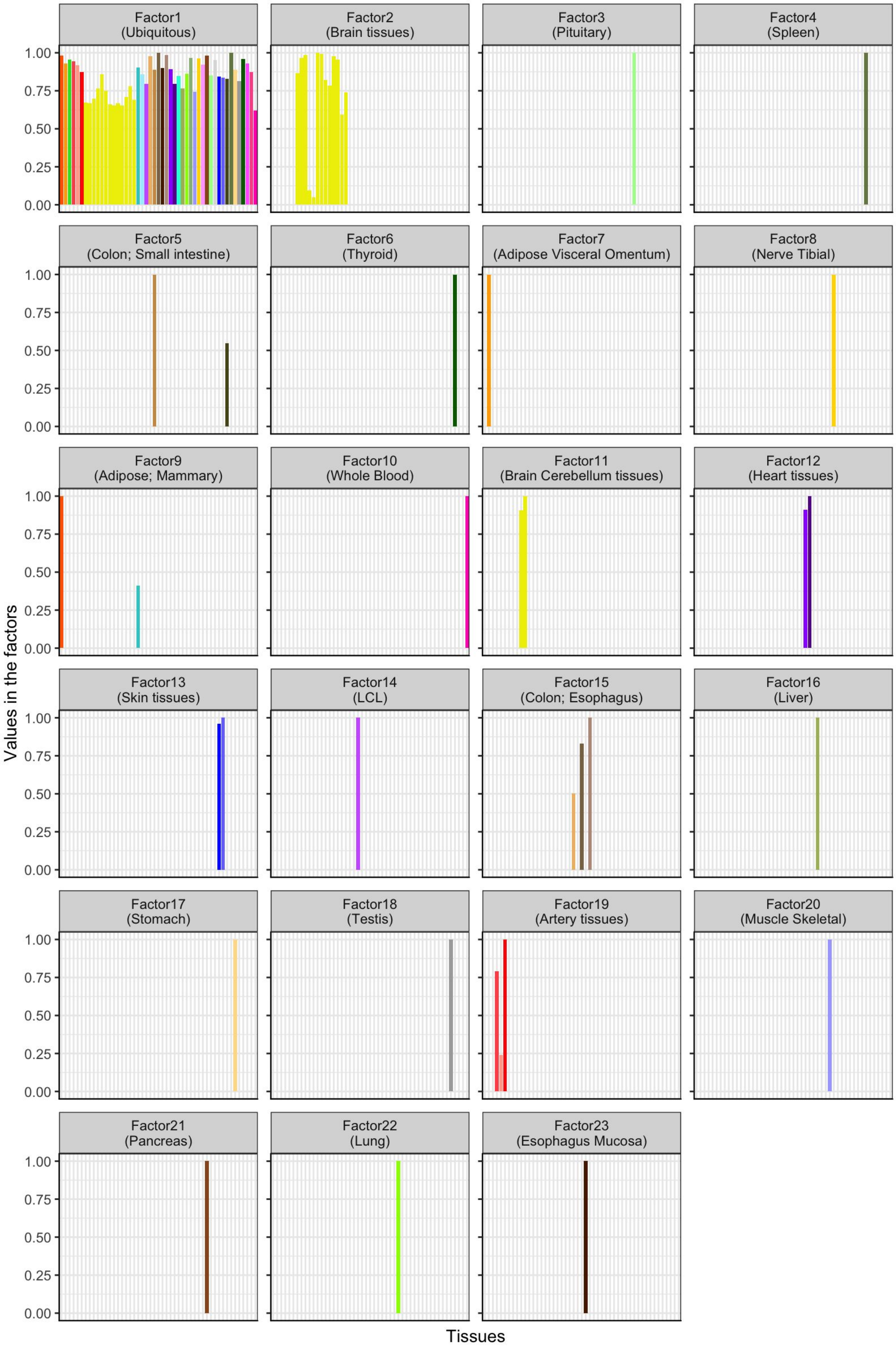

Figure S2. Learned factors from sn-spMF. Each panel represents one factor. Within each panel, x-axis represents the 49 tissues in GTEx, and y-axis represents the values in the factor for each tissue. Each factor is scaled to have maximum value = 1. The factors were named by the tissues with non-zero values in them.

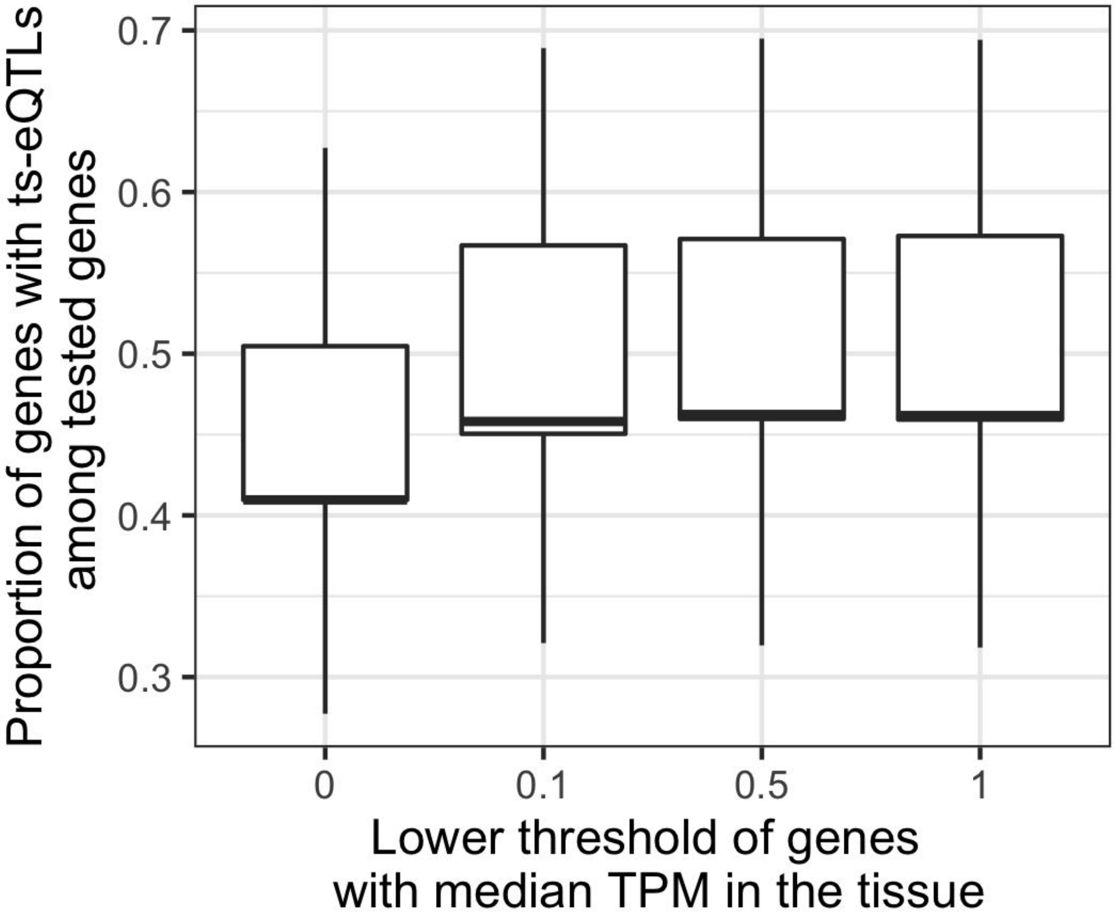

Figure S3. Proportion of genes with ts-eQTLs among tested genes after excluding genes with expression below varying TPM thresholds. In the boxplots, each dot represents the proportion of genes with ts-eQTLs in one tissue after excluding genes with median TPM lower than the threshold in that same tissue. Threshold = 0 means no threshold was used. Excluding genes with median expression below 0.1 rather than 0 results in a noticeable increase of the proportion of genes with ts-eQTLs. However, excluding additional genes with expression below other, higher thresholds results in only a marginal increase in the proportion of genes with ts-eQTLs. This indicates genes with moderately low expression do not exhibit a significant difference in the likelihood of being tissue-specific.

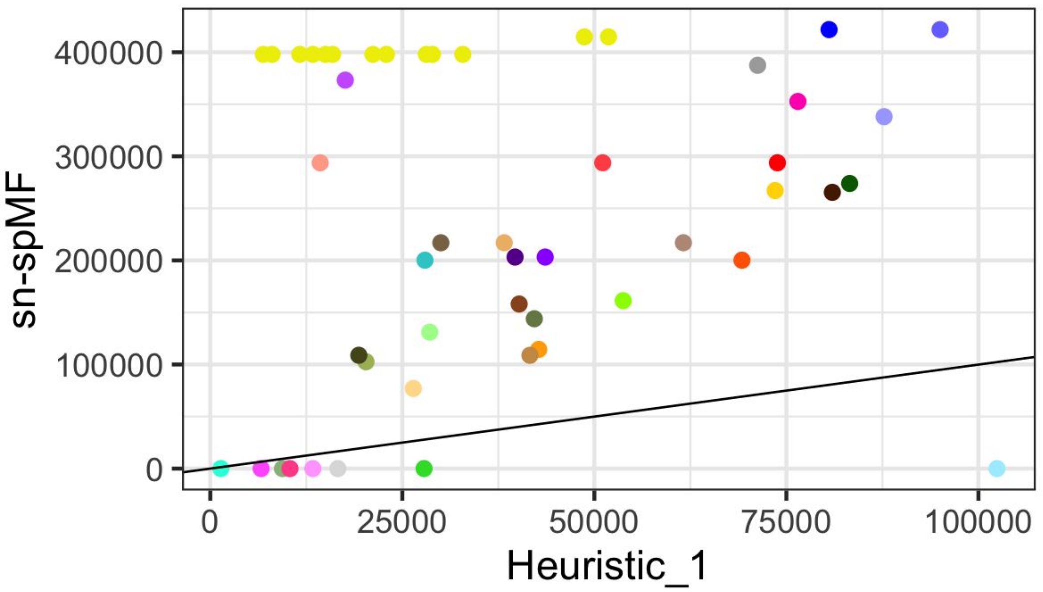

Figure S4. Comparison of the number of ts-eQTLs identified by heuristic_1 and sn-spMF. Each dot represents one tissue. X-axis represents the number of ts-eQTLs for each tissue from heuristic_1, and y-axis represents the number of ts-eQTLs for each tissue from sn-spMF. For sn-spMF, the number of ts-eQTLs for a tissue was computed by the number of eQTLs that load on tissue-specific factors with non-zero values for that tissue.

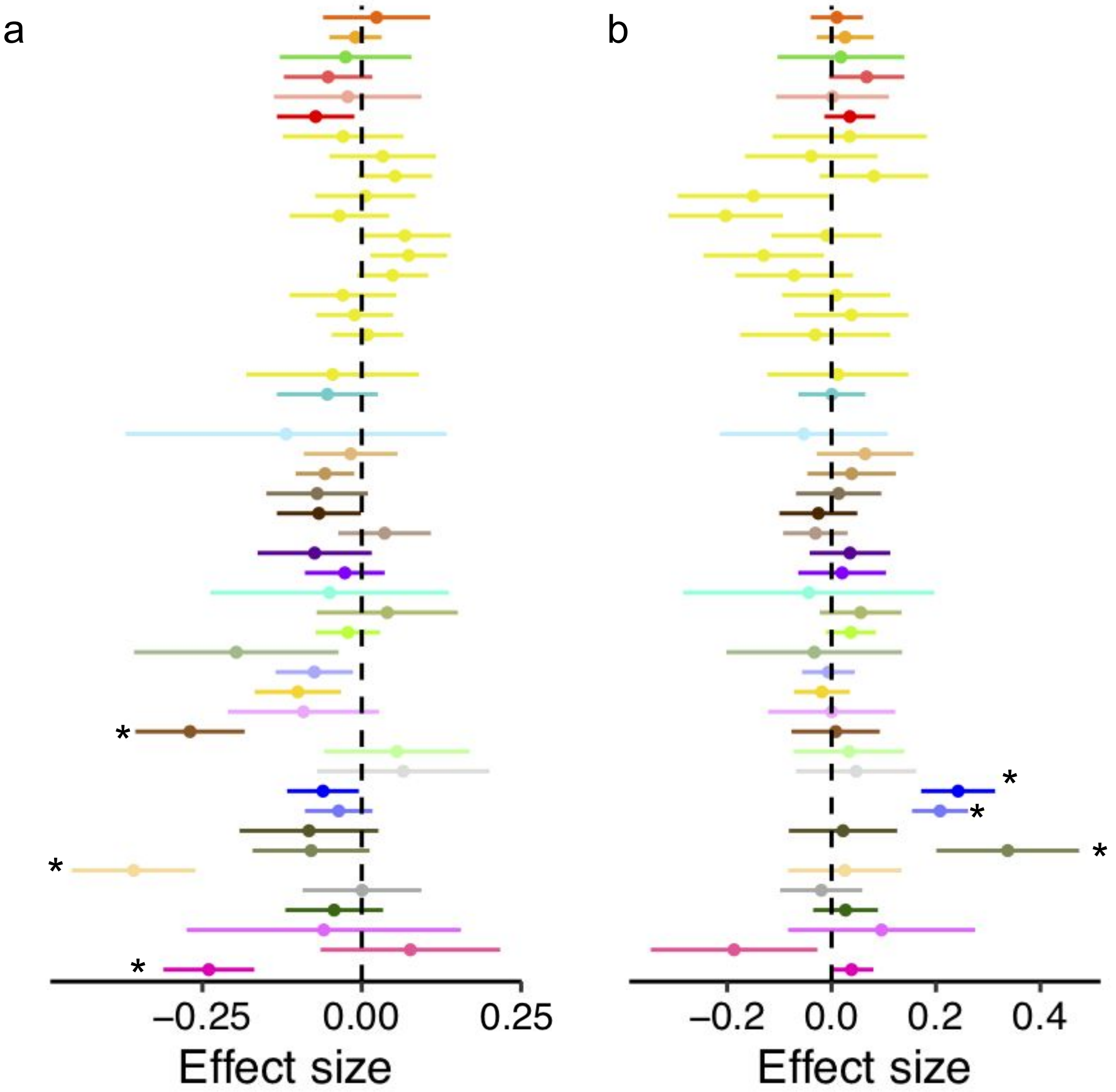

Figure S5. Examples of eQTLs identified by sn-spMF that have effects from multiple factors. Asterisk on the tissue indicates that this eQTL was significant with FDR < 0.05 in *that* tissue. **(a)** An eQTL (ENSG00000196730.12:chr9_87444328) with effects in the pancreas-specific factor, stomach-specific factor, and whole blood specific factor. **(b)** An eQTL (ENSG00000183655.12:chr15_85829822) with effects in the skin-specific factor and spleen-specific factor.

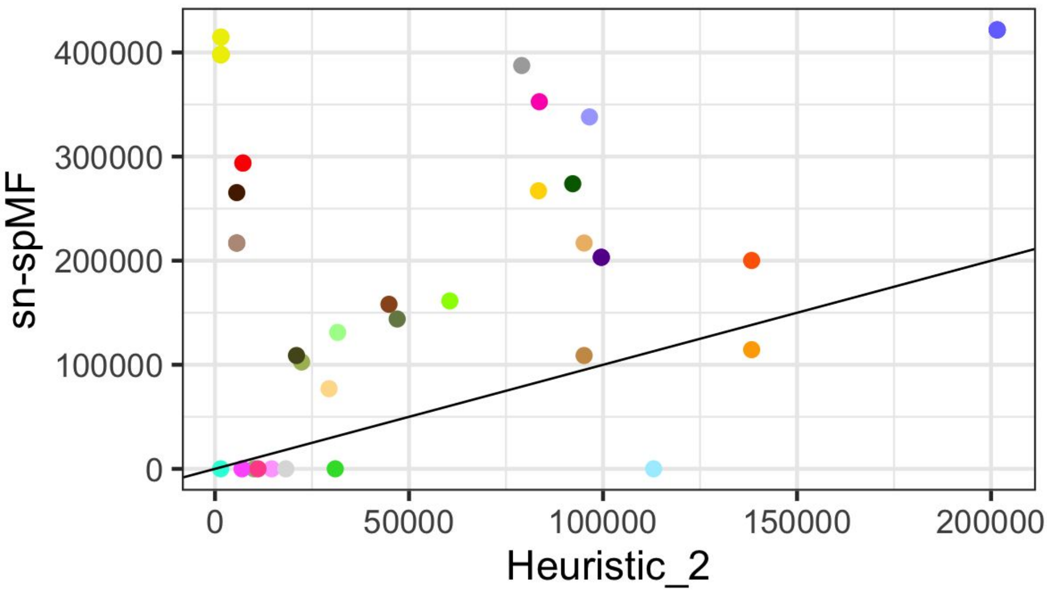

Figure S6. Comparison of the number of ts-eQTLs identified by heuristic_2 and sn-spMF. Each dot represents one tissue. X-axis represents the number of ts-eQTLs for each tissue from heuristic_1, and y-axis represents the number of ts-eQTLs for each tissue from sn-spMF. For sn-spMF, the number of ts-eQTLs for a tissue was computed by the number of eQTLs that load on tissue-specific factors with non-zero values for that tissue.

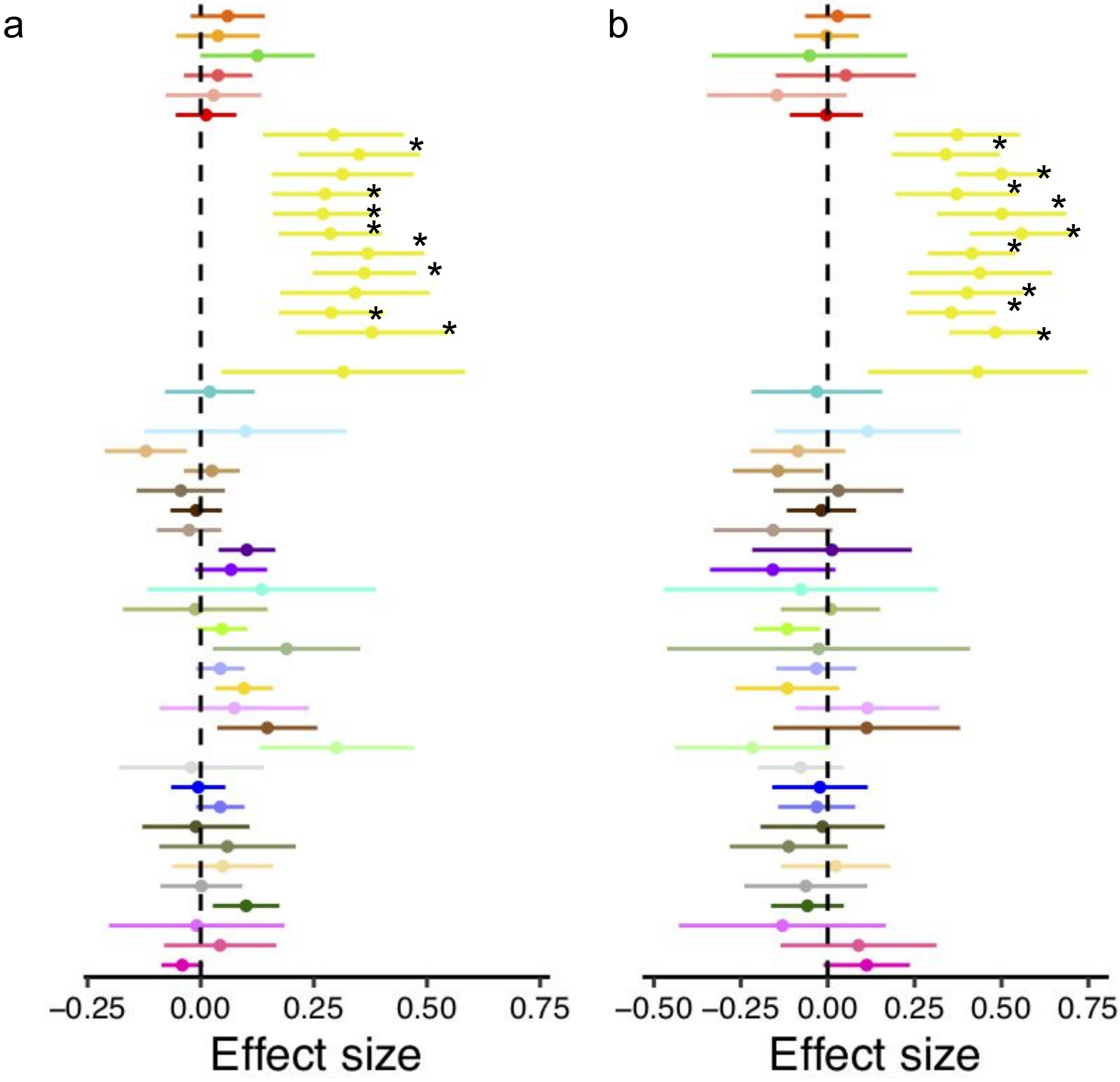

Figure S7. Examples of eQTLs identified as brain-specific eQTLs by heuristic_2, and not identified as ts-eQTL in any brain tissue by heuristic_1. Asterisk on the tissue indicates that this eQTL was tested to be significant in *that* tissue. **(a)** eQTL: ENSG00000009844.15:chr6_142169009 **(b)** eQTL: ENSG00000025423.11:chr12_56833972

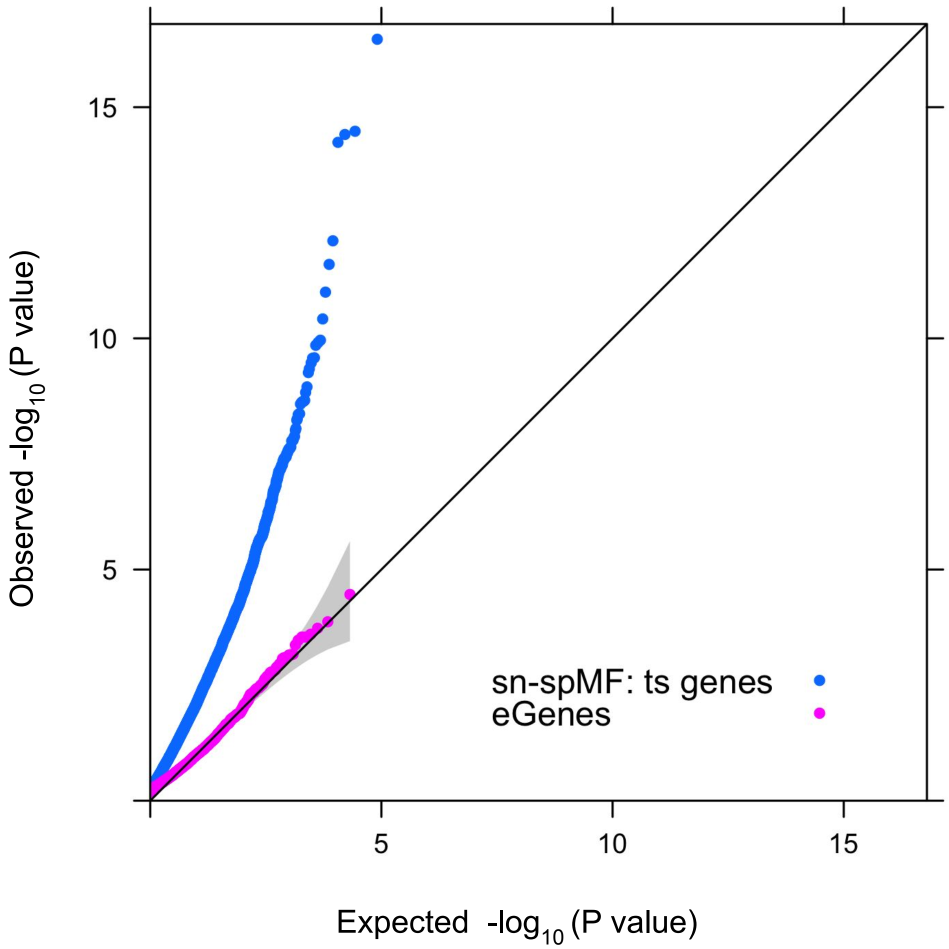

Figure S8. QQplot of $-\log_{10}$(P value) for gene set enrichment analysis (GSEA) for ts-eQTL genes from sn-spMF and eGenes for all the tissues. For each factor from sn-spMF, only the genes that were tested for the corresponding tissues were included as the background. For eGenes, genes tested for eQTL analysis were used as the background. We ran enrichment analysis using biological processes from the Gene Ontology (GO) project. At FDR < 0.05, 546 unique GO terms are enriched for tissue-specific genes, while no enrichment is found for the eGenes for each tissue. In the plot, each dot represents one data point at a specific quantile, with x-axis indicating $-\log_{10}$(P value) assuming that the P values are in uniform distribution, and y-axis indicating $-\log_{10}$(P value) of the true data point.

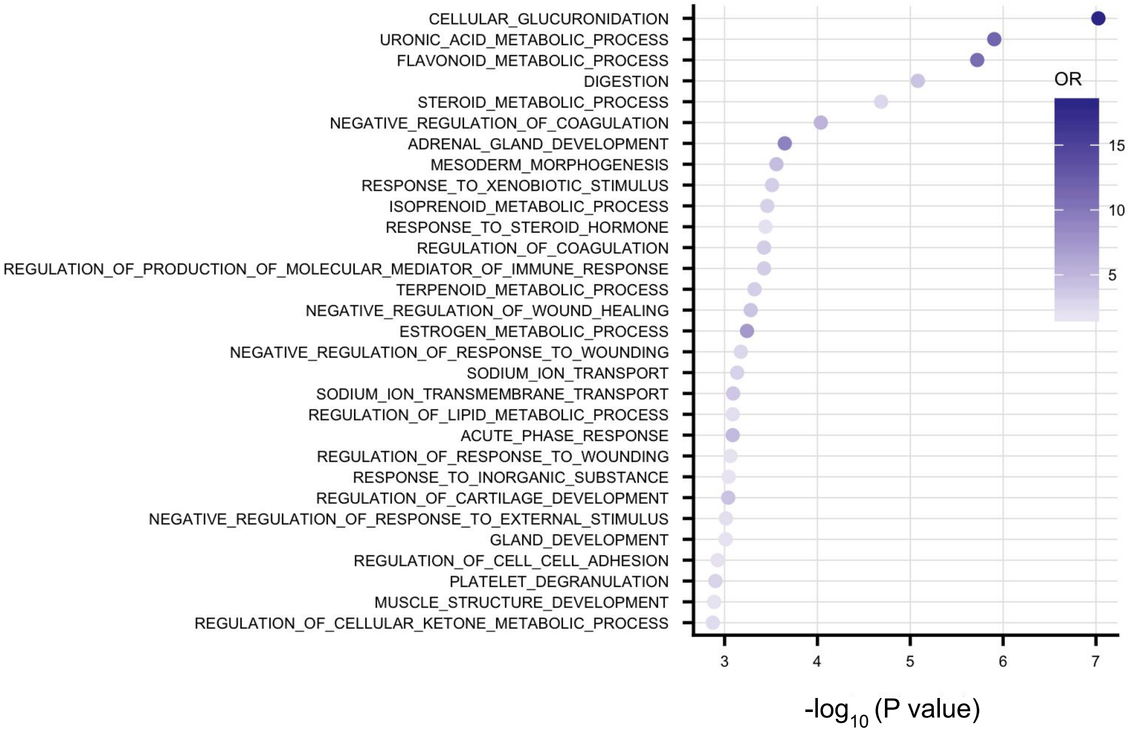

Figure S9. Top 30 enriched GO terms for genes with liver-specific eQTLs and no u-eQTL from sn-spMF. Gene set enrichment analysis was carried out comparing the odds of pre-defined liver-specific genes in a GO term to the odds of all tested genes from liver eQTL analysis in the GO term. X-axis shows $-\log_{10}$(P value) from fisher exact test of the enrichment analysis. Y-axis shows the top 30 enriched GO terms. The color bar represents the odds ratio (OR) from the enrichment analysis.

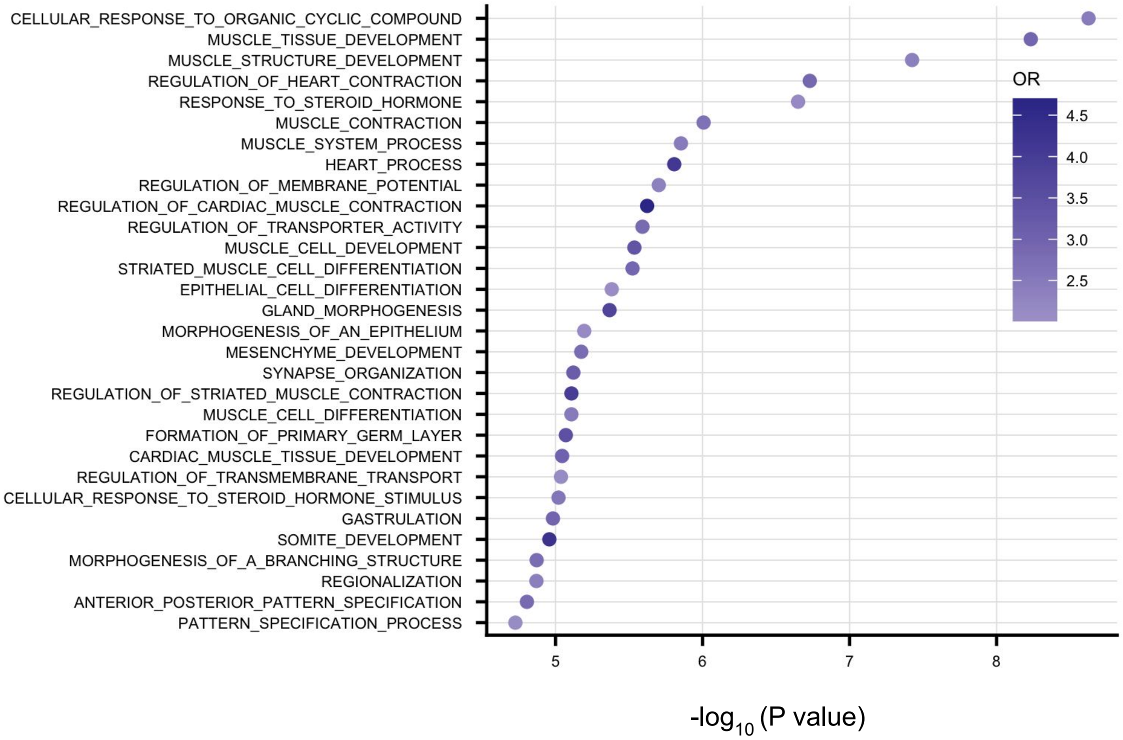

Figure S10. Top 30 enriched GO terms for genes with heart-specific eQTLs and no u-eQTL from sn-spMF

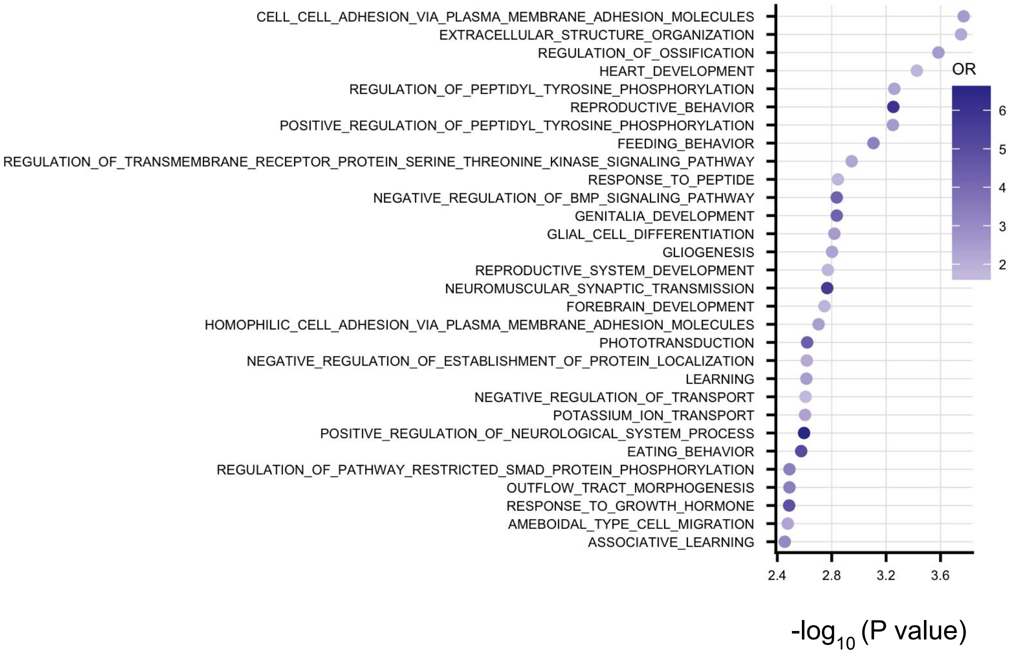

Figure S11. Top 30 enriched GO terms for genes with brain-specific eQTLs and no u-eQTL from sn-spMF

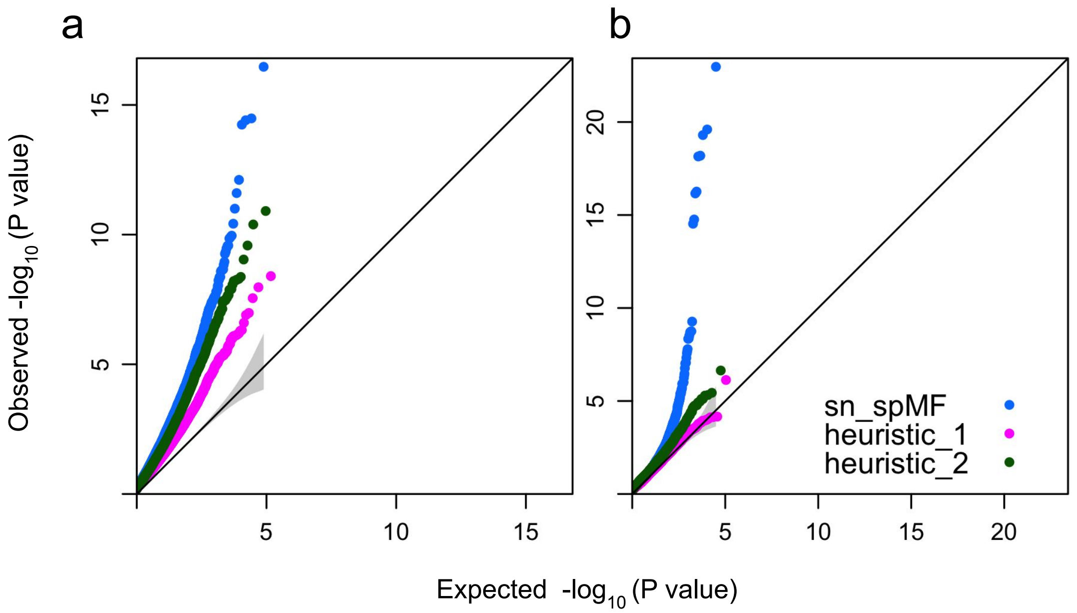

Figure S12. QQplot of -$\log_{10}$(P value) for tissue-specific eGenes from sn-spMF and two heuristic methods. We ran enrichment analysis using biological processes from the Gene Ontology (GO) project. Each dot represents one data point at a specific quantile, with x-axis indicating -$\log_{10}$(P value) assuming that the P values are in uniform distribution, and y-axis indicating -$\log_{10}$(P value) of the true data point. **(a)** Results using tissue-specific eGenes with ts-eQTLs and with no u-eQTL. **(b)** Results using tissue-specific eGenes that appear in less than X factors (X = 6 for sn-spMF, X = 7 for heuristic_1, X = 6 for heuristic_2 for comparable size of the gene sets). At FDR < 0.05, 45 GO terms are enriched for sn-spMF, no GO term is enriched for heuristic_1, and one GO term is enriched for heuristic_2.

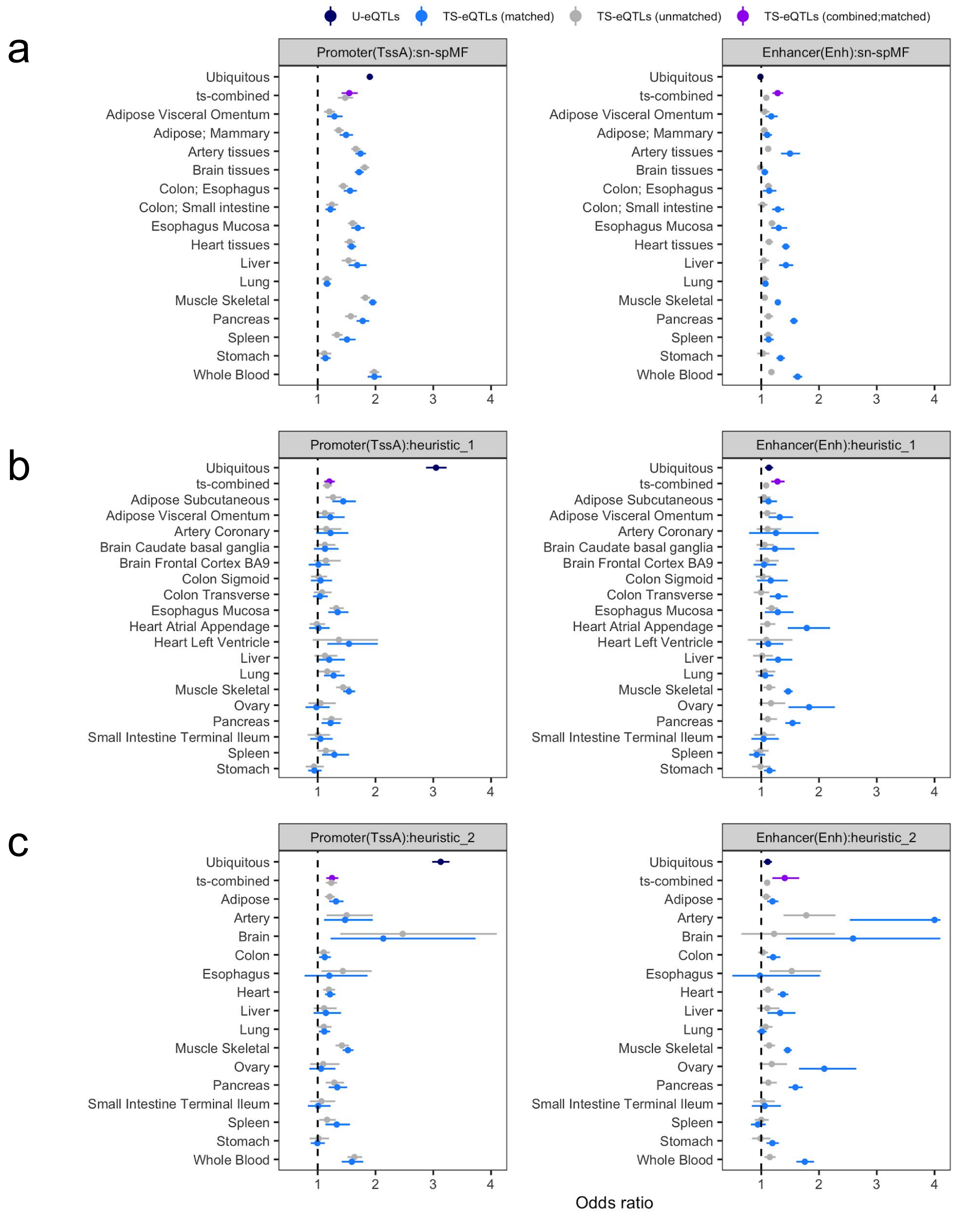

Figure S13. Enrichment analysis of chromatin state annotations for u-eQTL variants and ts-eQTL variants from sn-spMF, and two heuristic methods. eQTL types presented along the y-axis, Odds ratio of enrichment relative to random SNPs matched for MAF and distance to gene TSS (see Methods) presented along the x-axis **(a)** For sn-spMF, ts-eQTL variants are more enriched in enhancers than u-eQTL variants (OR=1.3 and OR=1.0), while u-eQTL variants are more enriched in promoters than ts-eQTL variants (OR=1.9 and OR=1.5) . **(b)** For heuristic_1, ts-eQTL variants are more enriched in enhancers than u-eQTL variants (OR=1.3 and OR=1.1), while u-eQTL variants are more enriched in promoters than ts-eQTL variants (OR=3.0 and OR=1.2) . **(c)** For heuristic_2, similar to heuristic_1, ts-eQTL variants are more enriched in enhancers than u-eQTL variants (OR=1.4 and OR=1.1), while u-eQTL variants are more enriched in promoters than ts-eQTL variants (OR=3.1 and OR=1.2) .

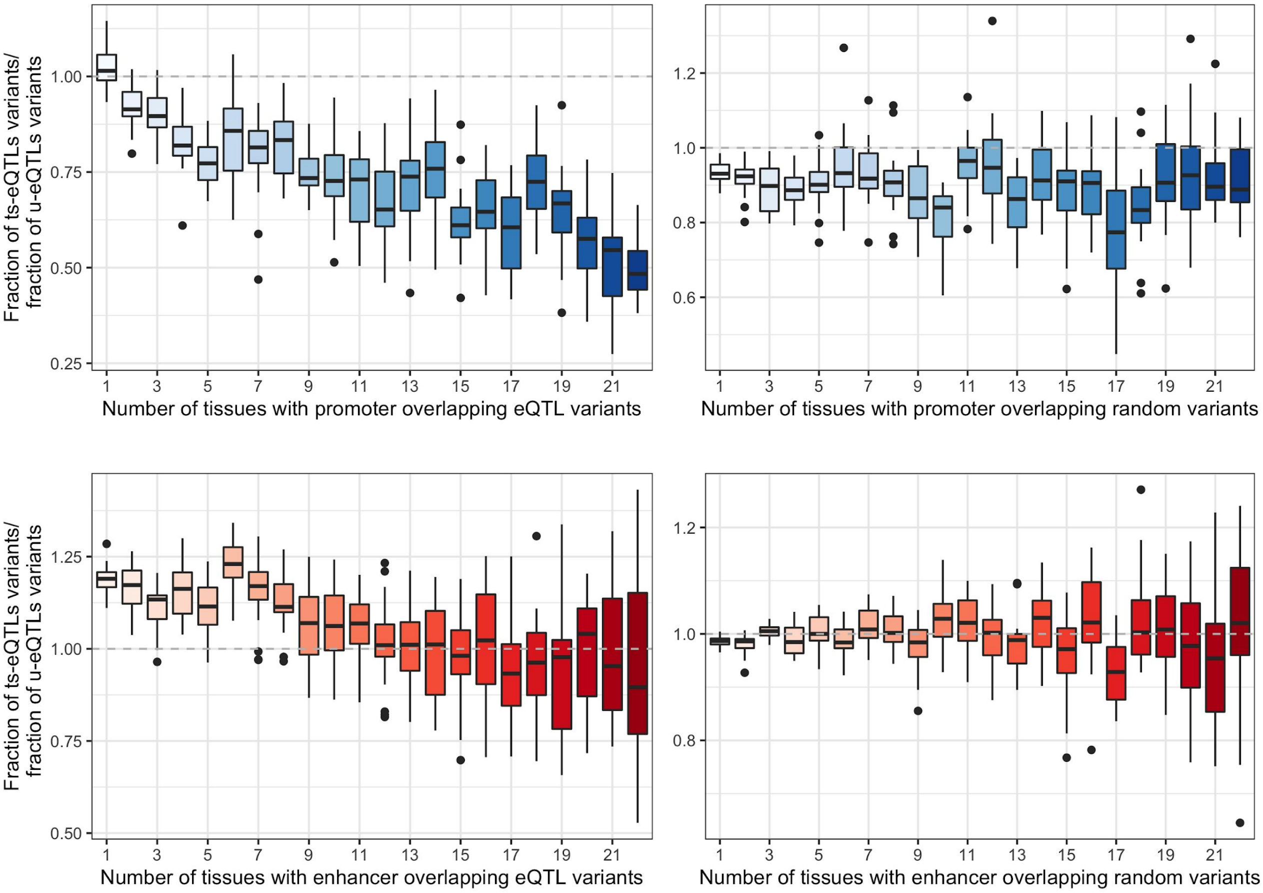

Figure S14. The proportion of ts-eQTL variants that are located in enhancer/promoter elements active in different numbers of tissues, scaled by the proportion of u-eQTL variants in the corresponding class of element. ChromHMM annotation "7_Enh" was used to indicate enhancers, and "1_TssA" was used to indicate promoters. In each boxplot, the normalized proportion of ts-eQTLs in each bin is shown on the y-axis, while the number of tissues with an enhancer/promoter overlap is shown on the x-axis. The same analysis is repeated for random SNPs matched for of u-eQTLs and ts-eQTLs. The random SNPs are matched for MAF and distance to gene transcription start sites (TSS). Unlike random SNPs, ts-eQTLs are less likely to overlap promoters shared across many tissues. Furthermore, ts-eQTLs are more likely to overlap enhancers in only a small number of tissues compared to u-eQTLs (left bottom panel, x-axis 1-8).

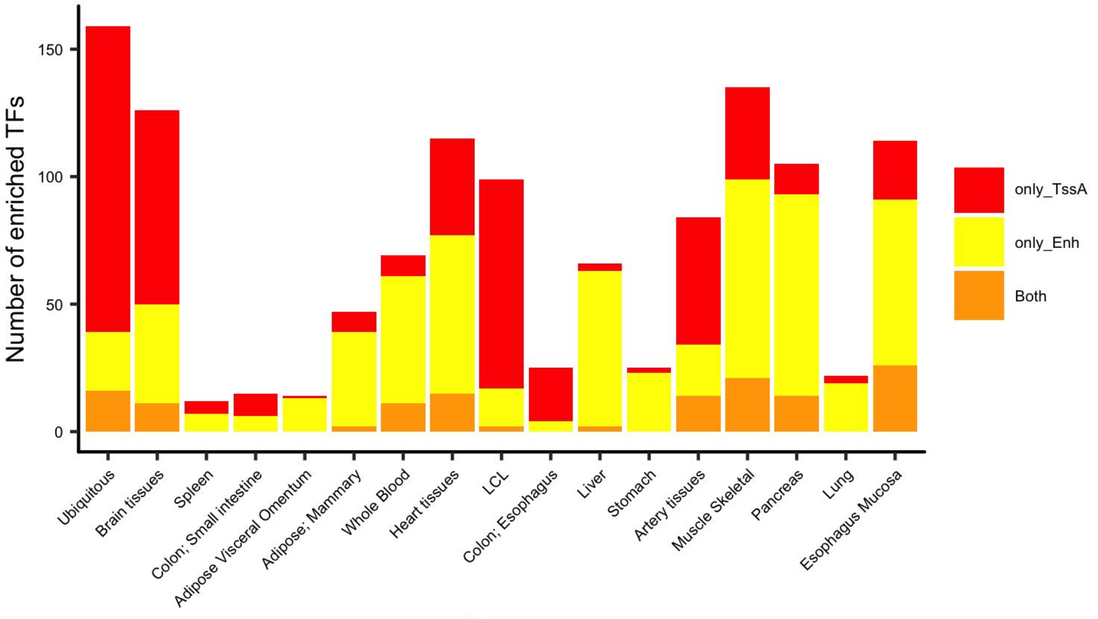

Figure S15. TFs specifically enriched in only promoters, or only in enhancers, or in both promoters and enhancers for eQTLs across factors from sn-spMF. X-axis represents the factors, and y-axis represents the number of enriched TFs for eQTLs that are loaded on each factor.

a

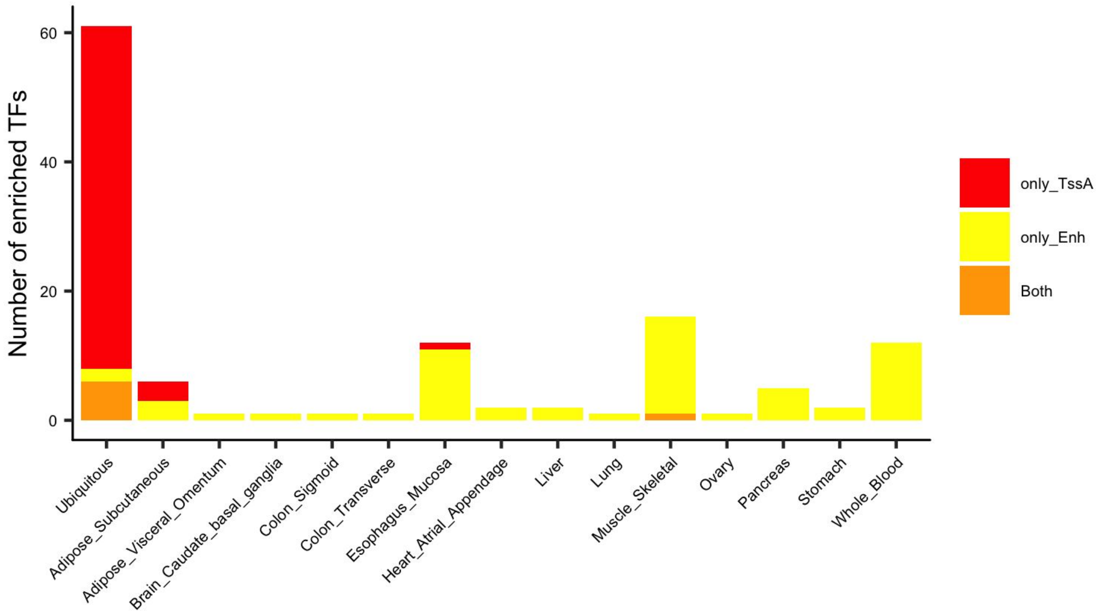

b

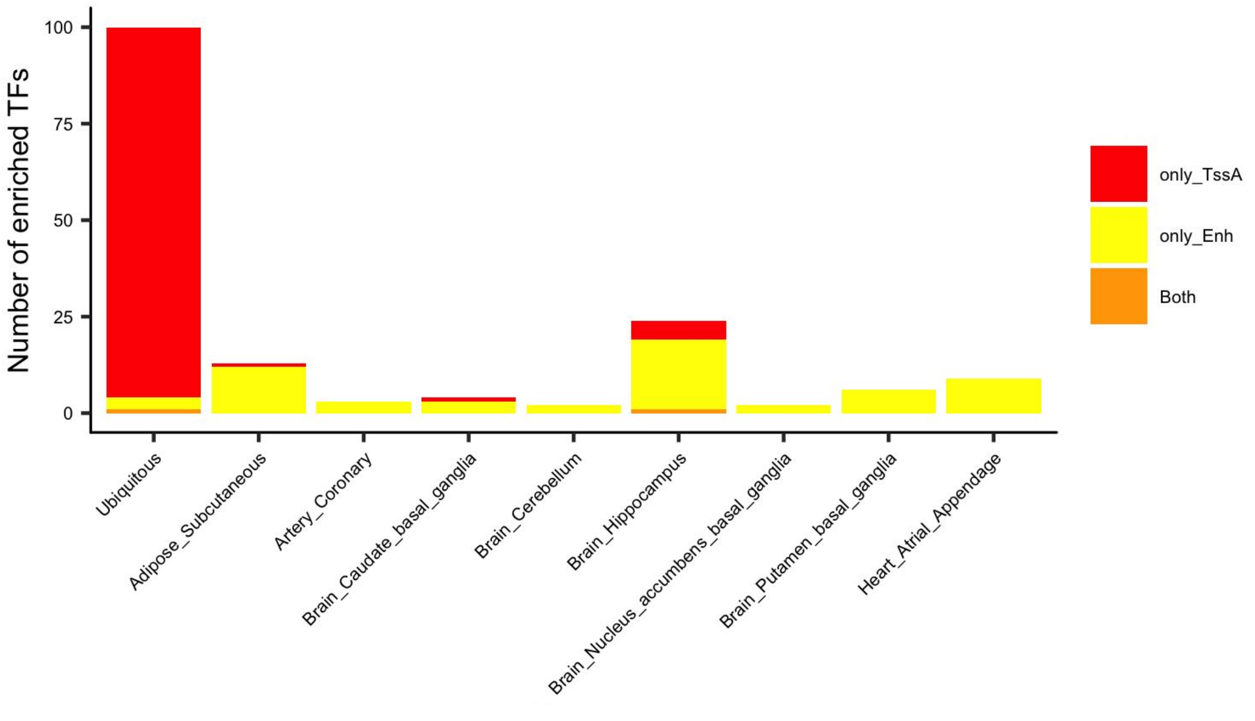

Figure S16. Number of enriched TFs in in enhancers and promoters for eQTLs **(a)** from heuristic_1 and **(b)** from heuristic_2. X-axis represents the factors, and y-axis represents the number of enriched TFs for eQTLs that are loaded on each factor.

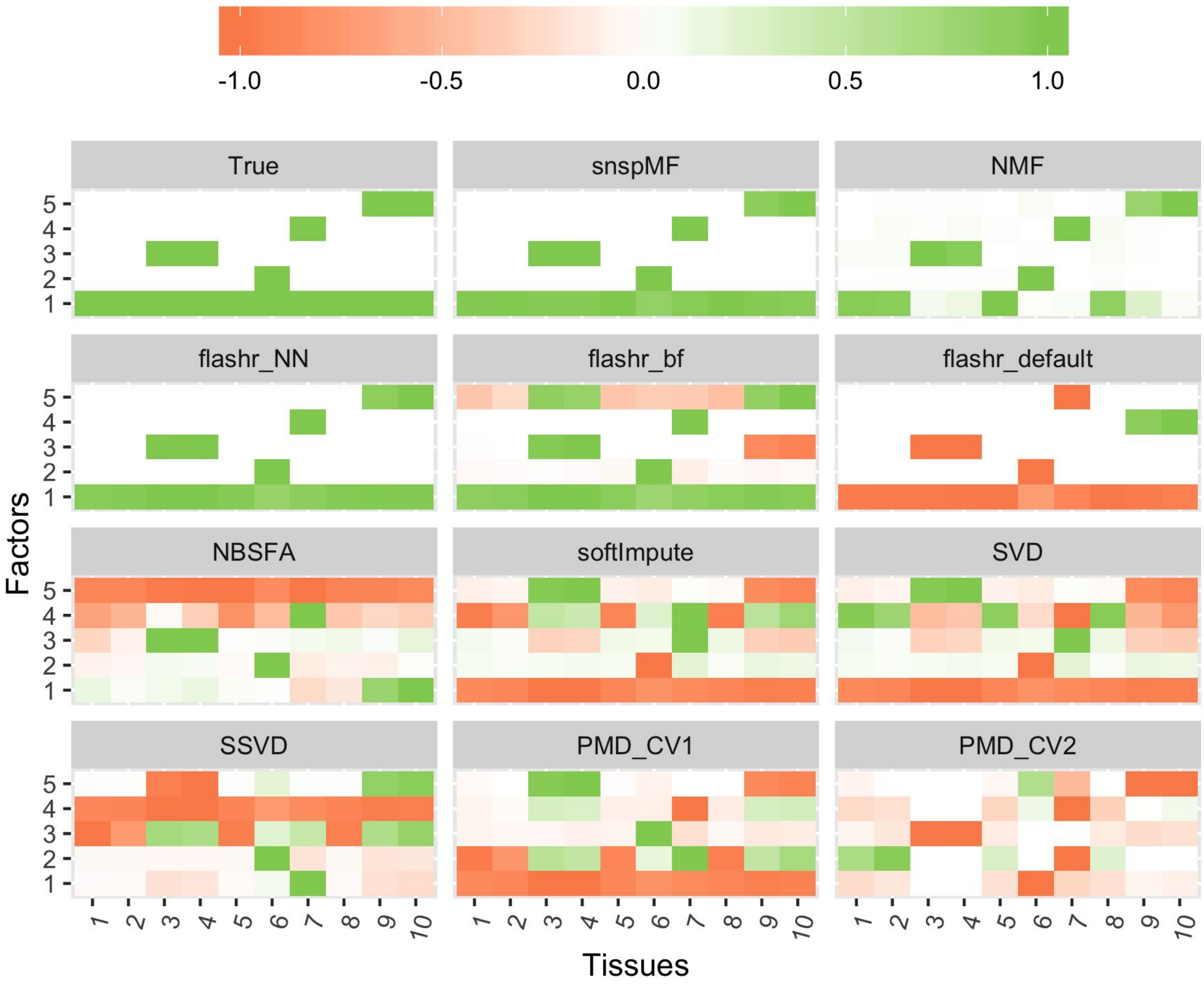

Figure S17. Fitted matrices of simulated data using a variance of error matrix of 0.01 in one round of simulation. The color bar shows the values in the factors, with green representing positive values, white representing zeros, and red representing negative values.

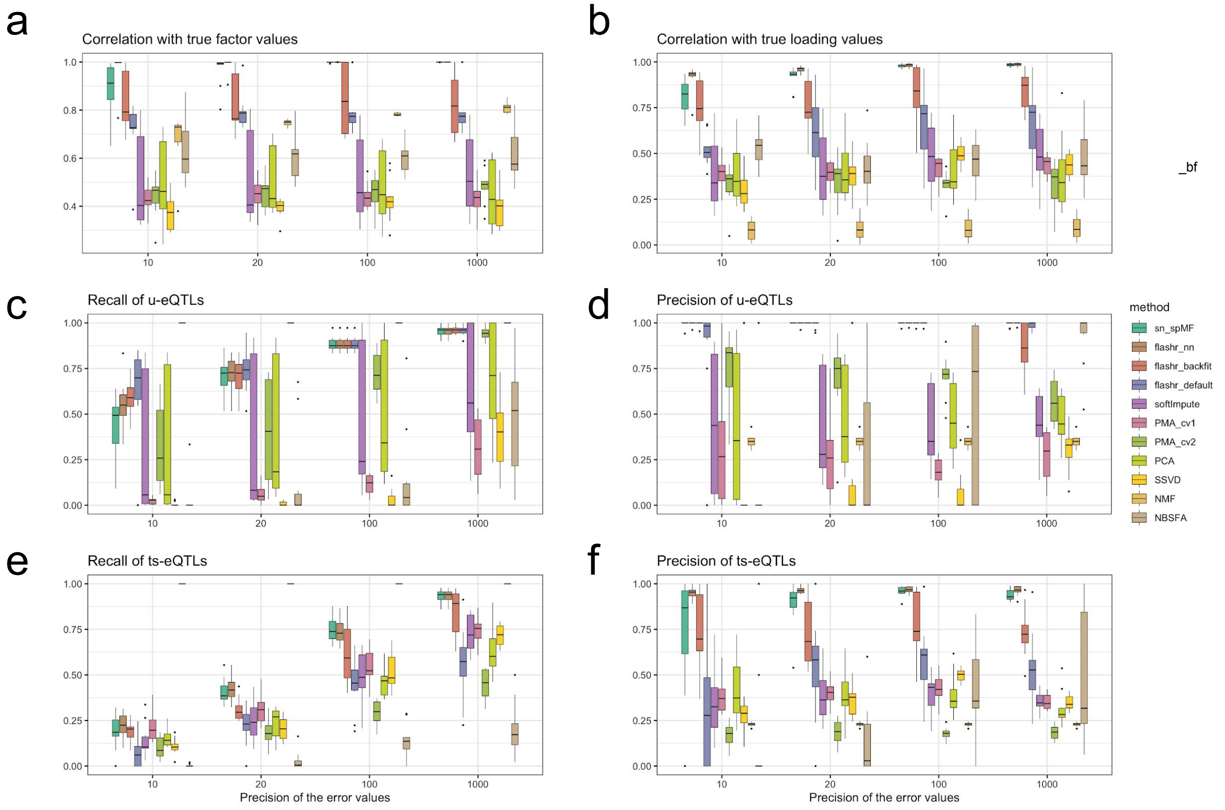

Figure S18. Performance of multiple matrix factorization methods. Each box includes results from 10 random simulations. X-axis represents the precision of the error values, and y-axis presents (a) absolute correlation between true and learned factors; (b) absolute correlation between true and learned loadings; (c) recall of u-eQTLs, computed as the proportion of u-eQTLs that are identified as u-eQTL; (d) precision of u-eQTLs, computed as the proportion of called u-eQTLs that are truly u-eQTL; (e) recall of ts-eQTLs, computed as the proportion of ts-eQTLs that are identified as ts-eQTL; (d) precision of ts-eQTLs, computed as the proportion of called ts-eQTLs that are truly ts-eQTL.

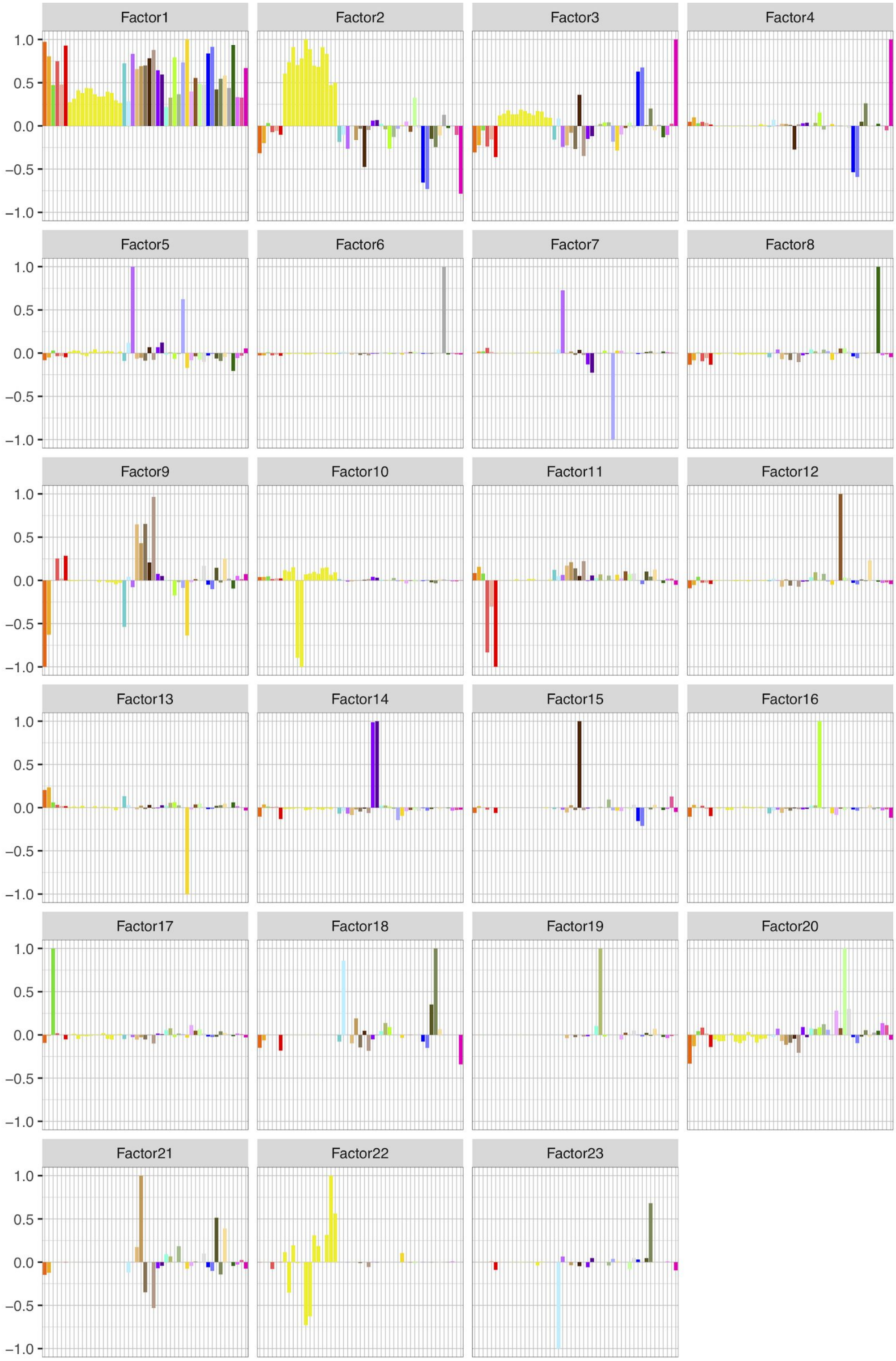

Figure S19. Factor matrix learned from flashr_bf using the same input eQTLs as sn-spMF. Flashr was fit using *flash_greedy_workhorse* and *flash_backfit_workhorse* (see Methods). Each panel represents one factor. Within each panel, x-axis represents the 49 tissues in GTEx, and y-axis represents the values in the factor for each tissue. Each factor is scaled to have maximum absolute value = 1.

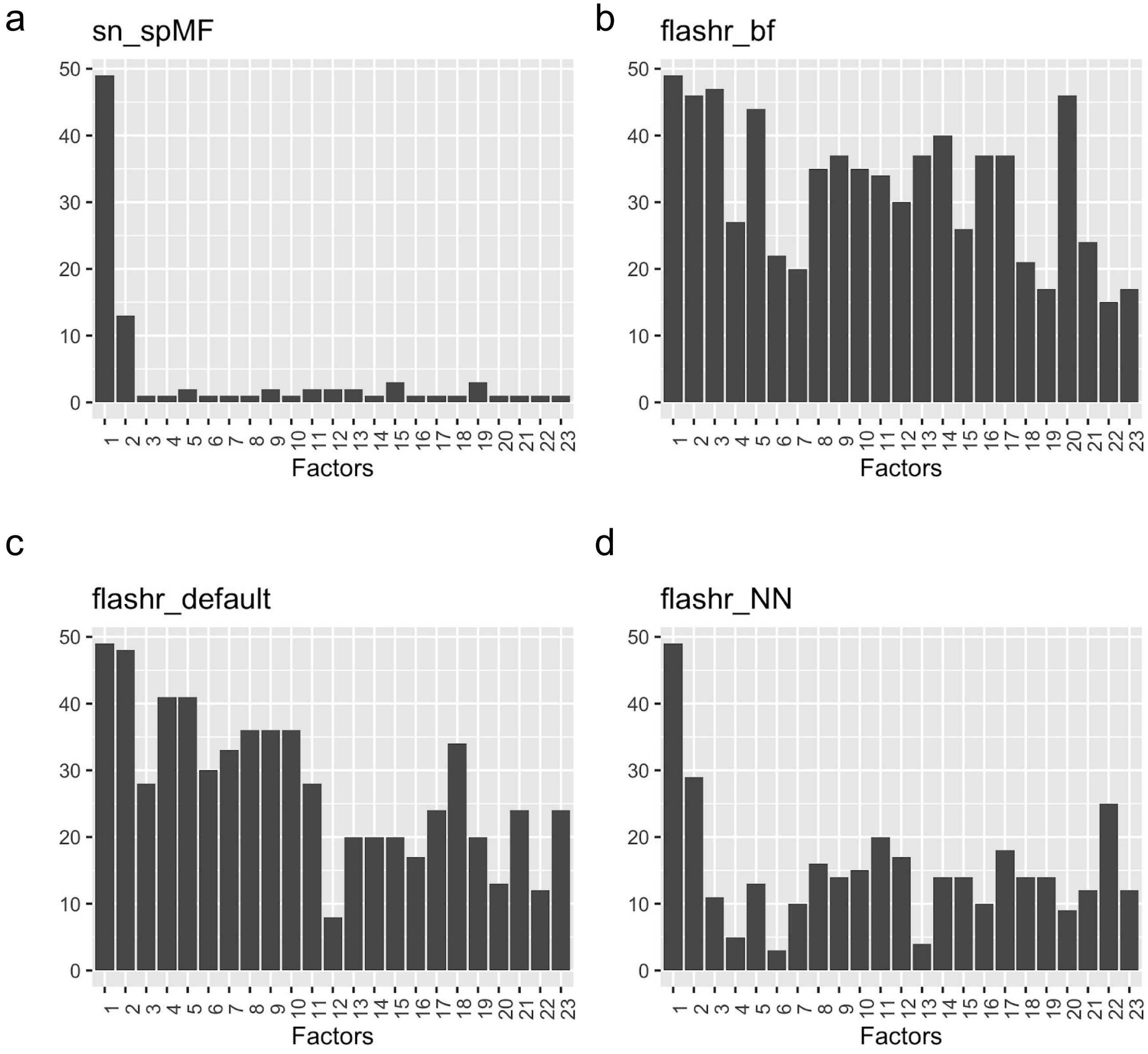

Figure S20. Sparsity for the learned factors. X-axis represents the factors, and y-axis represents number of tissues with absolute value > 0.01 in each factor that is scaled to have absolute maximum value = 1. Each panel shows the results for a MF method: **(a)** sn-spMF; **(b)** flashr with backfitting; **(c)** default flashr; **(d)** flashr with non-negative prior.

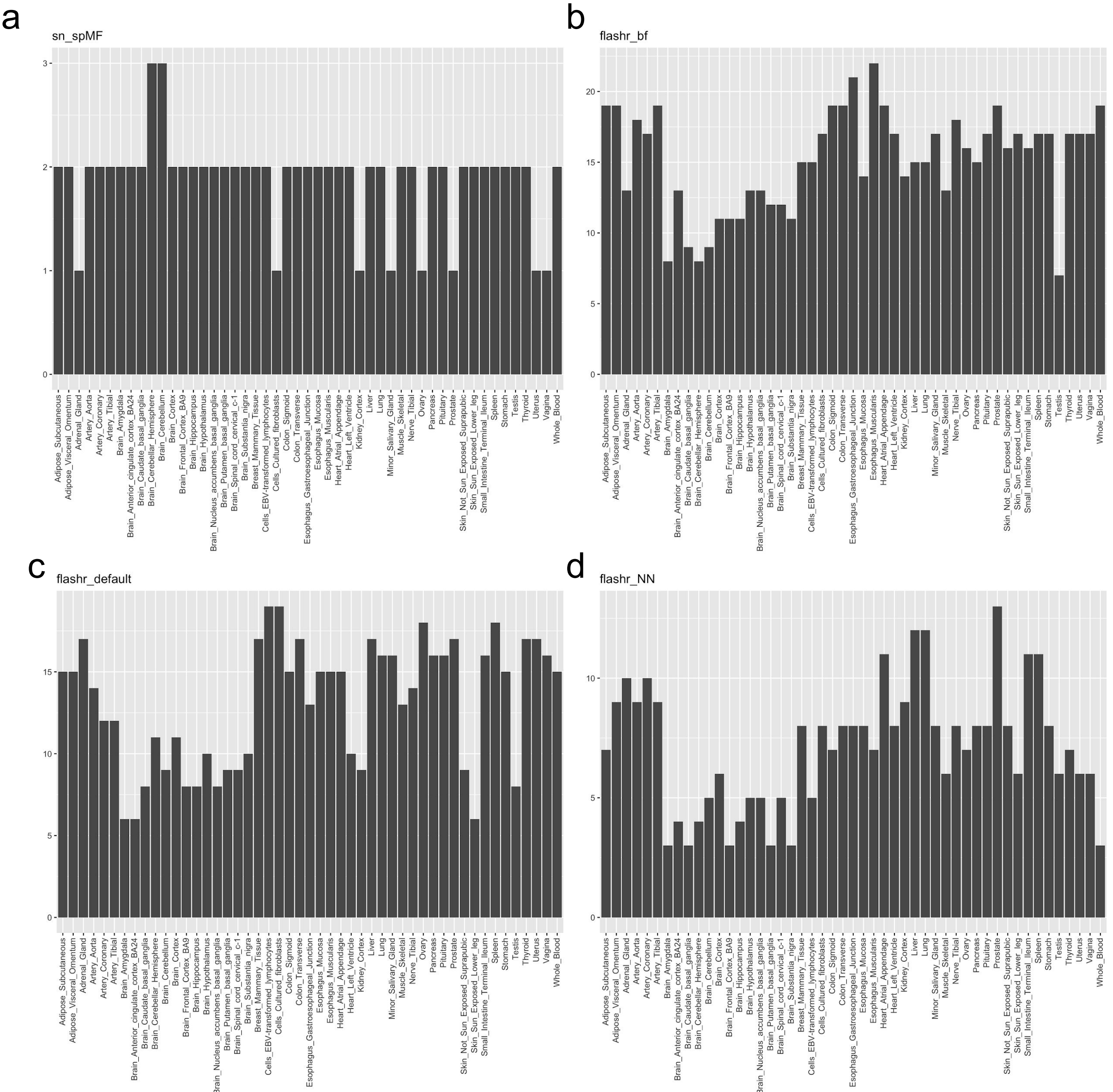

Figure S21. Sparsity for the learned factors. X-axis represents the tissues, and y-axis represents number of factors where a tissue has absolute value > 0.01 (factors are scaled to have absolute maximum value = 1). Each panel shows the results for a MF method: **(a)** sn-spMF; **(b)** flashr with backfitting; **(c)** default flashr; **(d)** flashr with non-negative prior.

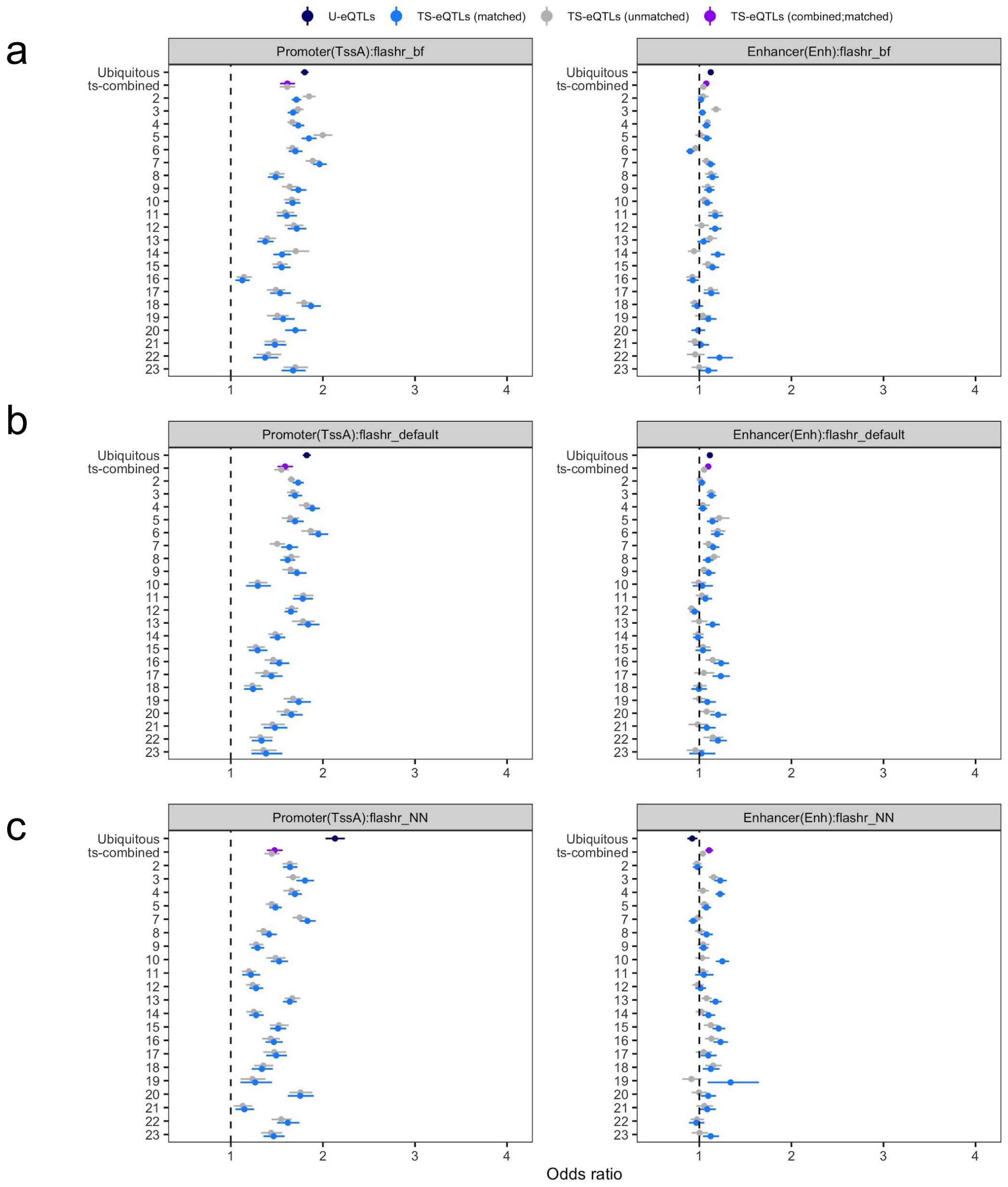

Figure S22. Enrichment analysis of cis-regulatory regions for u-eQTL variants and ts-eQTL variants from flashr with backfitting (flashr_bf), default flashr (flashr_default), and flashr with a non-negative prior (flashr_NN). Y-axis represents eQTL types or the factor eQTLs are loaded on. X-axis represents odds ratio of enrichment relative to random SNPs matched for MAF and distance to gene TSS (see Methods) **(a)** For flashr_bf, u-eQTL and ts-eQTL variants are similarly weakly enriched for enhancers (OR=1.1 and OR=1.1). **(b)** For flashr_default, both u-eQTL and ts-eQTL variants are both weakly enriched for enhancers, with ts-eQTLs not demonstrating greater enrichment than u-eQTLs (OR=1.1 and OR=1.1). **(c)** For flashr_NN, u-eQTL variants are more enriched in promoters than ts-eQTL variants (OR=2.1 and OR=1.5), while ts-eQTLs are more enriched in enhancers than u-eQTLs (OR=1.1 and OR =0.9). Also, there is slightly increased OR for ts-eQTLs in enhancers in the matched tissues compared to the OR in enhancers in unmatched tissues.

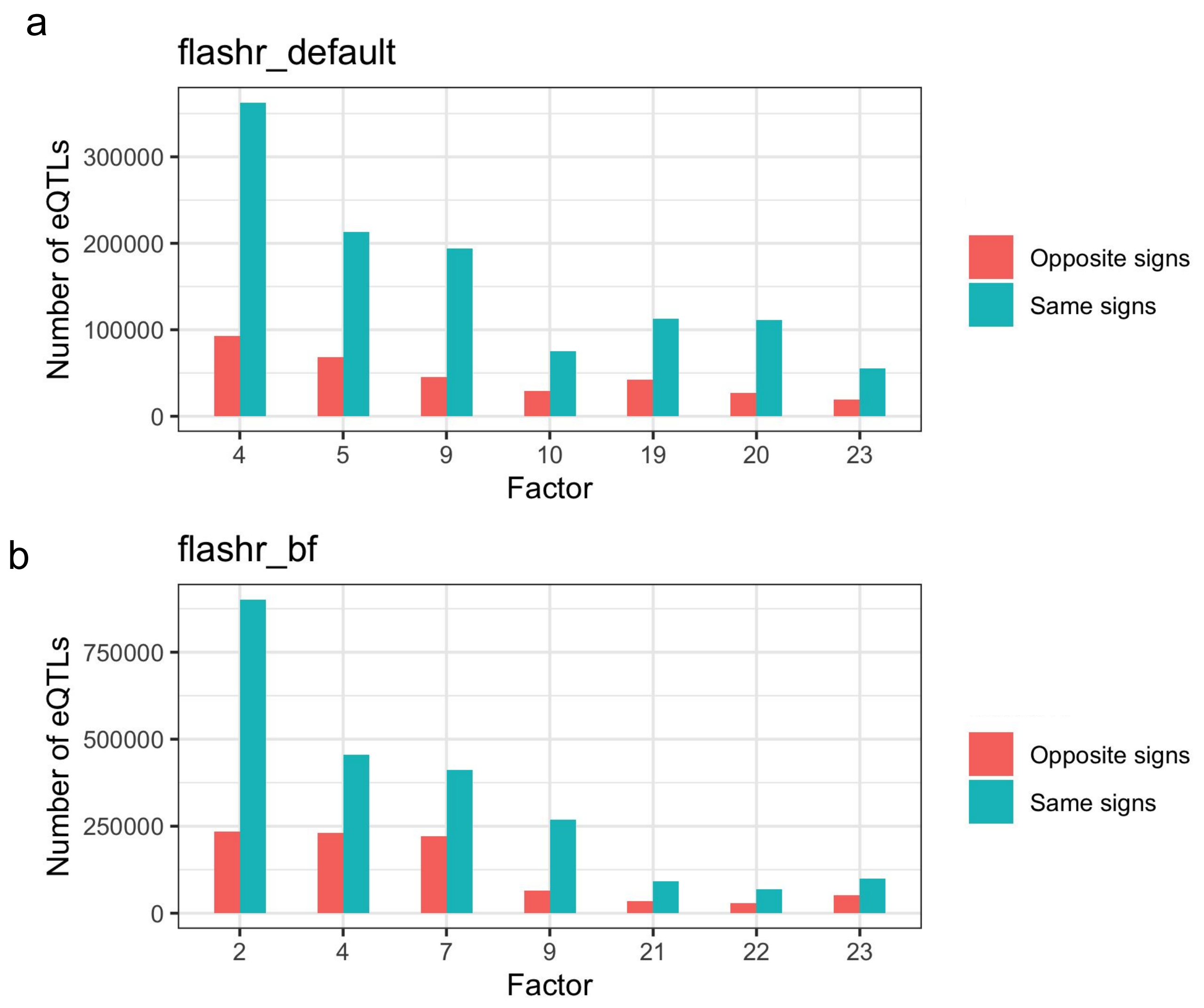

Figure S23. Evaluation of flashr factors with mixed sign across tissues. The x-axis represents the factors where tissues have large effects in opposite directions (absolute value > 0.5). Red bars represent number of eQTLs assigned to each factor that have opposite signs in the tissues with opposite values. If there is more than one tissue with opposite value, the tissue with the most extreme Z-score is used. Blue bars represent the number of eQTLs assigned to each factor that have the same signs. There are 19% - 28% eQTLs with opposite signs for flashr_default (**a**), and 19% - 35% eQTLs with opposite signs for flashr_backfitting (**b**).

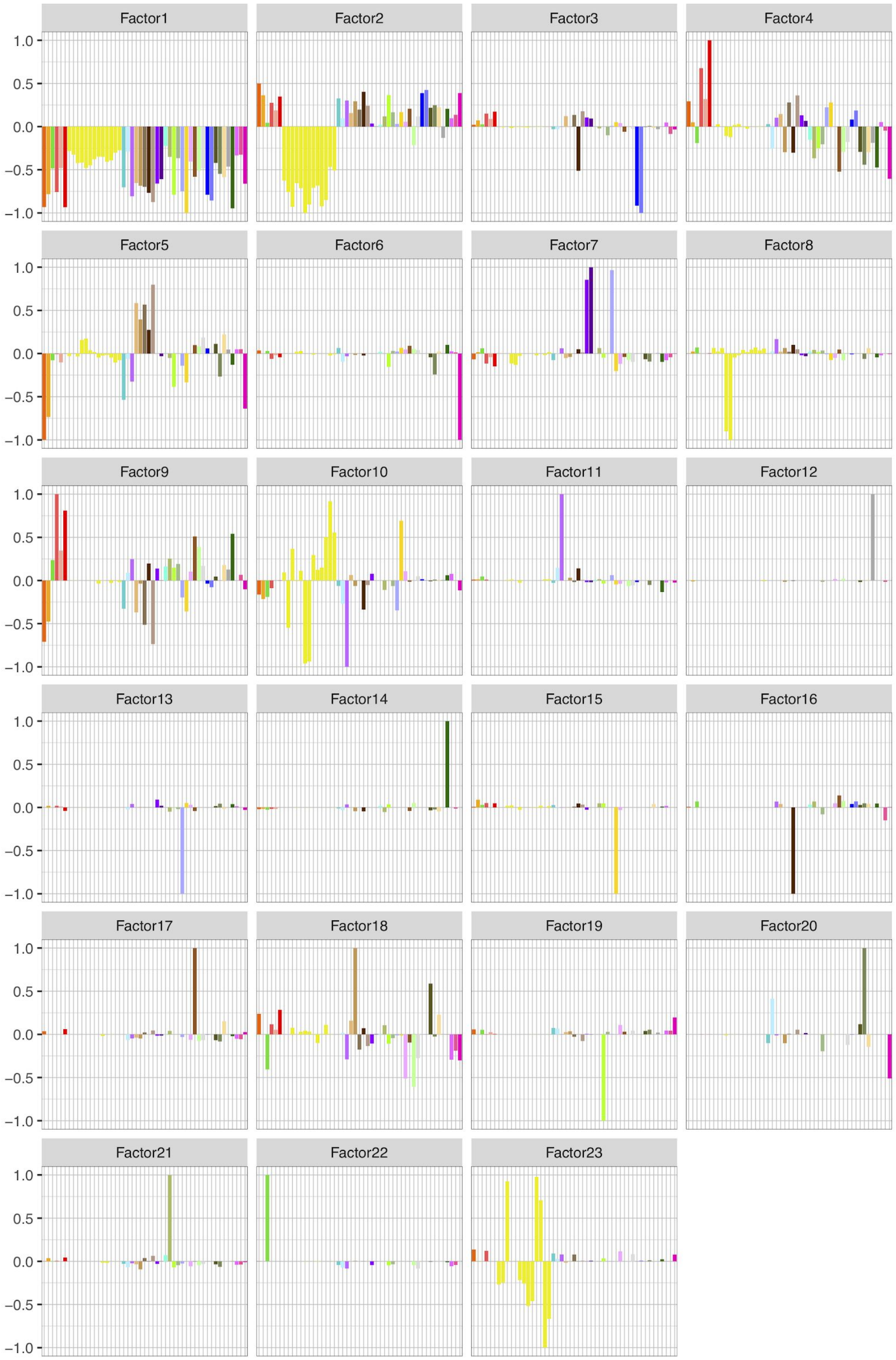

Figure S24. Factor matrix learned from default flashr using the same input eQTLs as sn-spMF. Flashr was fitted using *flashr*. Each panel represents one factor. Within each panel, x-axis represents the 49 tissues in GTEx, and y-axis represents the values in the factor for each tissue. Each factor is scaled to have maximum absolute value = 1.

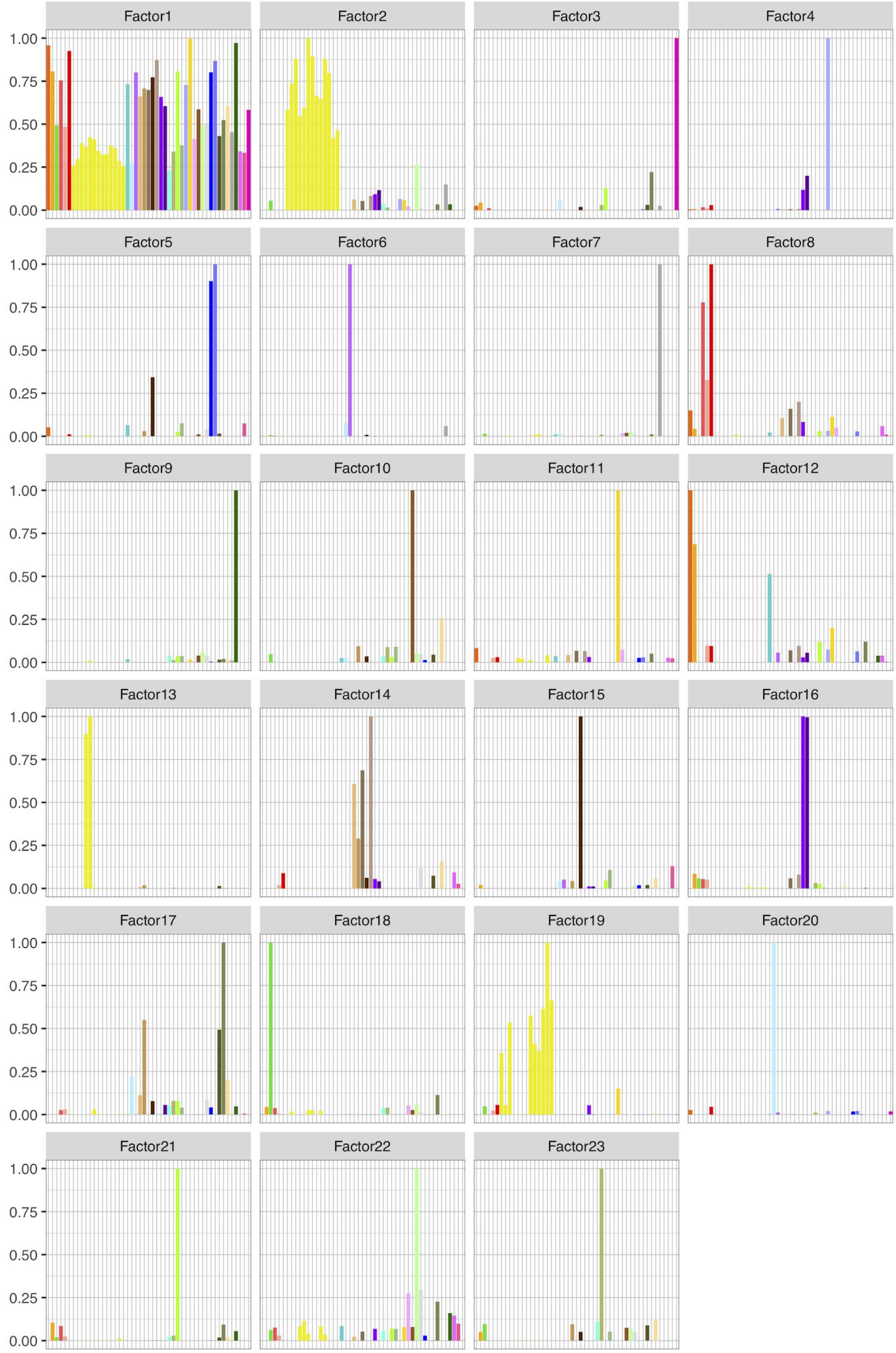

Figure S25. Factor matrix learned from flashr_NN using the same input eQTLs as sn-spMF. Flashr was fit using *flash_greedy_workhorse* and *flash_backfit_workhorse*, with non-negative priors on the factors. Each panel represents one factor. Within each panel, x-axis represents the 49 tissues in GTEx, and y-axis represents the values in the factor for each tissue. Each factor is scaled to have maximum absolute value = 1. (to be continued.)

Figure S25. (continue the legend) We observed high multicollinearity of the flashr_NN ubiquitous factor with the tissue-specific factors, which made it difficult to distinguish tissue-specific effects across numerous factors from truly ubiquitous effects. This is indicated by *variance inflation factor* of the ubiquitous factor VIF = 1/(1 - R2), where R2 denotes the coefficient of determination obtained by fitting a regression model for the ubiquitous factor on all other factors. We observed a very high VIF of 46 for flashr_NN, compared to 1.5 for flashr_default and 4.5 for sn-spMF, where guidelines suggest that a VIF higher than 10 may lead to imprecise estimates of the regression coefficients (Yoo et al. 2014, Stine 1998)

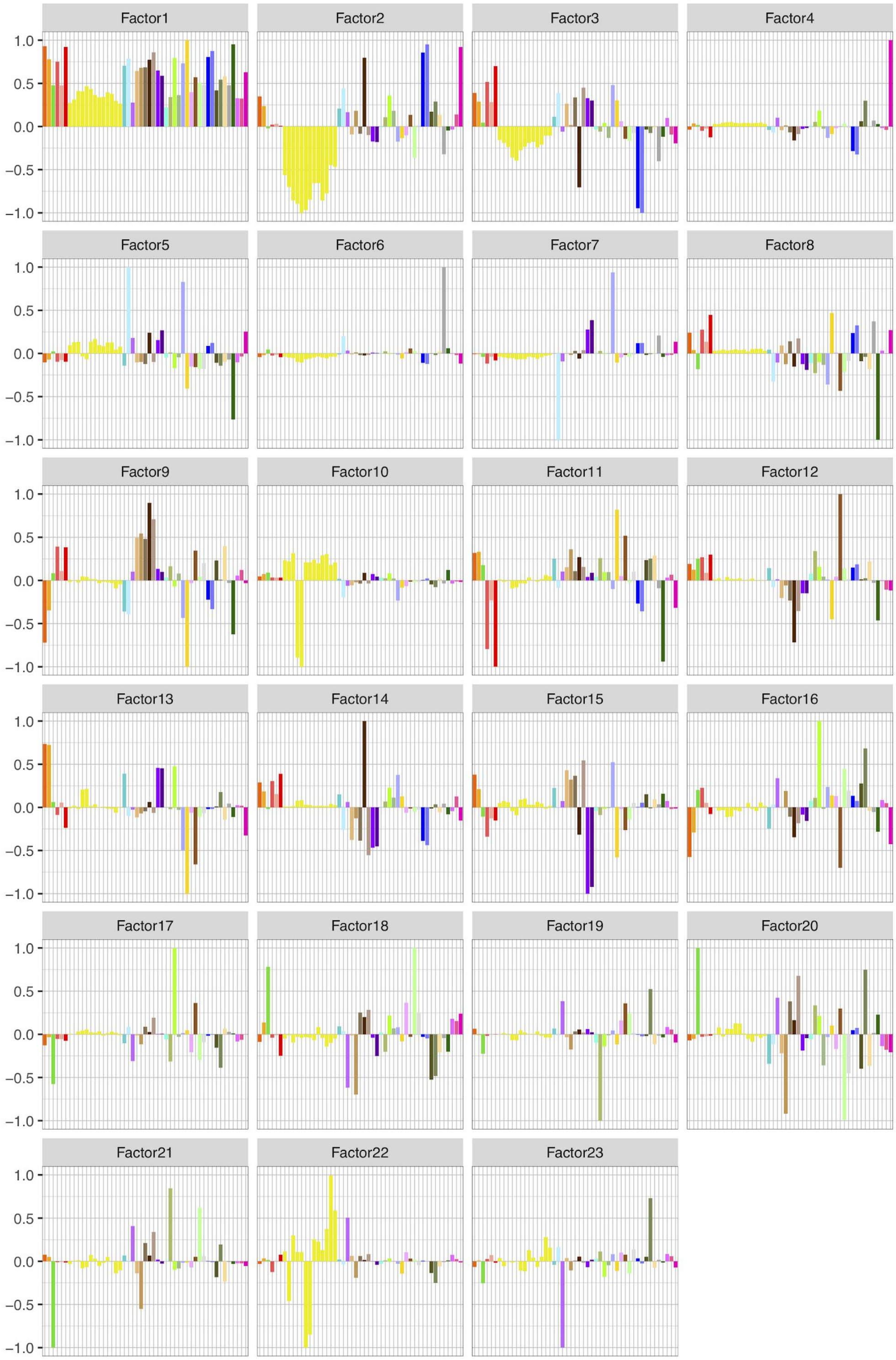

Figure S26. Factor matrix learned from softImpute. Each panel represents one factor. Within each panel, x-axis represents the 49 tissues in GTEx, and y-axis represents the values in the factor for each tissue. Each factor is scaled to have maximum absolute value = 1. The factors are more dense (few non-zero entries, each factor reflecting a large set of tissues) compared to factors learned by sn-spMF, and are thus more difficult to interpret in terms of tissue-specificity

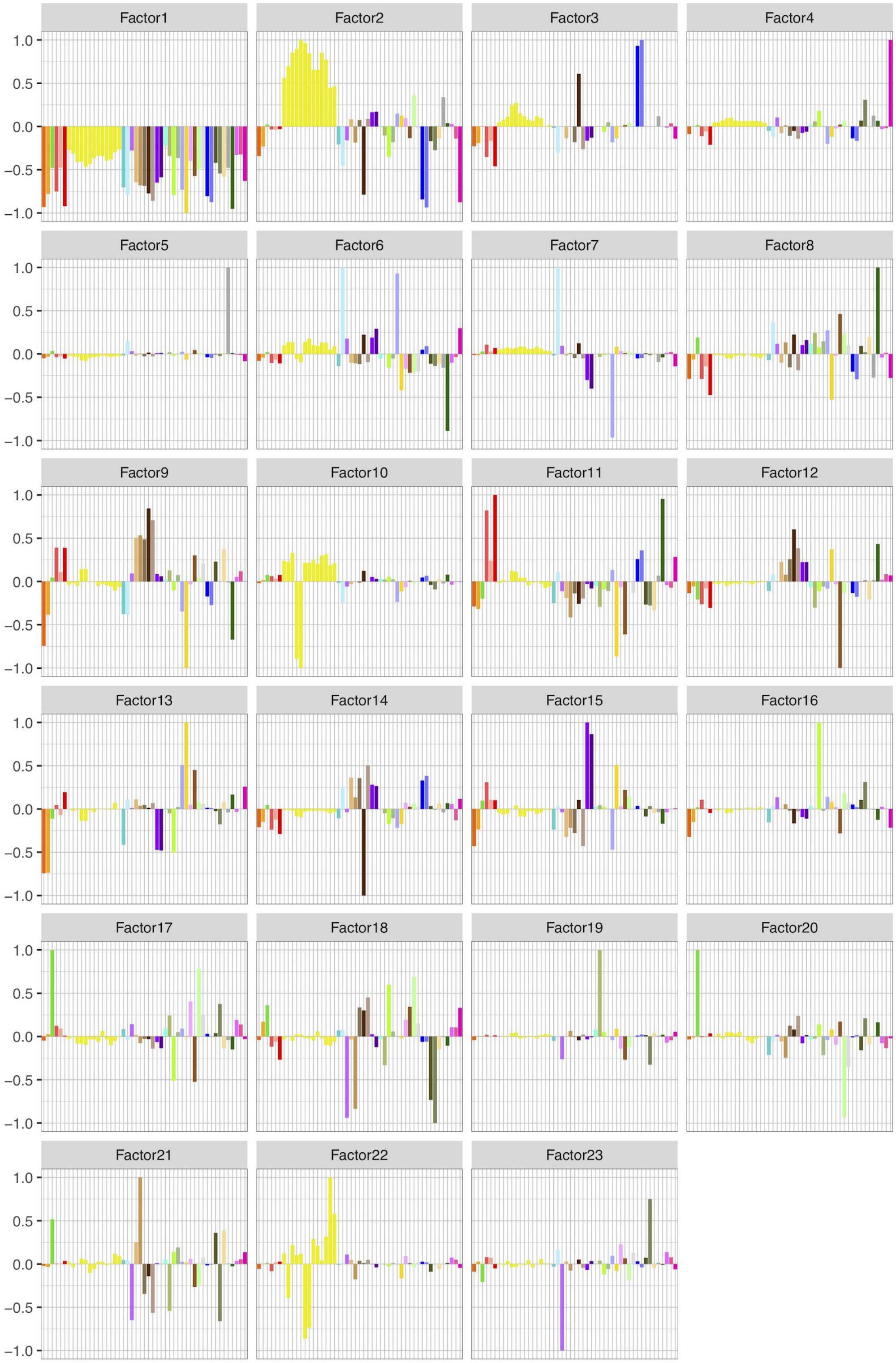

Figure S27. Factor matrix learned from PMD_cv1. Each panel represents one factor. Within each panel, x-axis represents the 49 tissues in GTEx, and y-axis represents the values in the factor for each tissue. Each factor is scaled to have maximum absolute value = 1. Similarly, factor are more dense compared to factors learned by sn-spMF, and are thus more difficult to interpret in terms of tissue-specificity

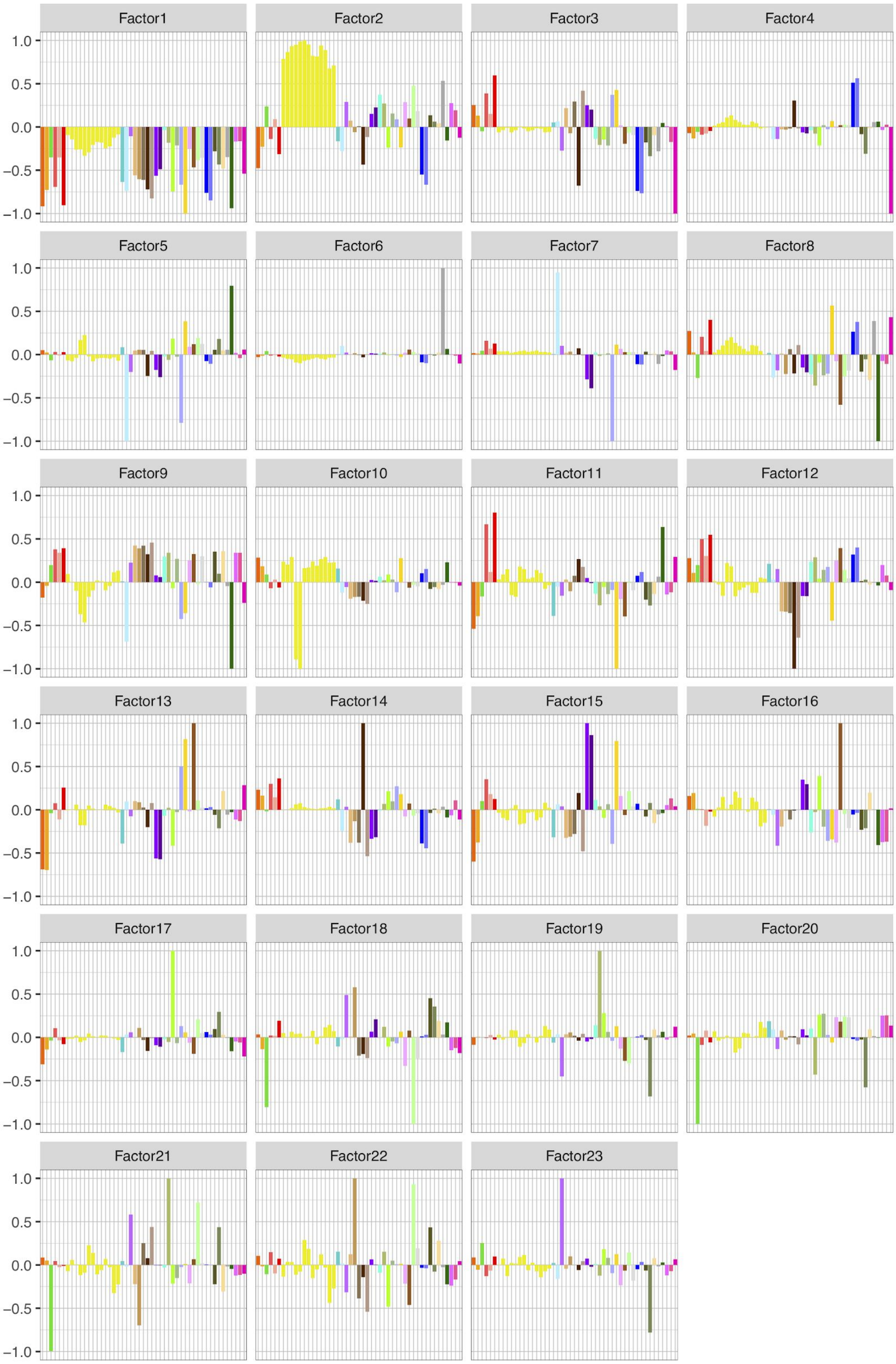

Figure S28. Factor matrix learned from PMD_cv2. Each panel represents one factor. Within each panel, x-axis represents the 49 tissues in GTEx, and y-axis represents the values in the factor for each tissue. Each factor is scaled to have maximum absolute value = 1. Similarly, factor are more dense compared to factors learned by sn-spMF, and are thus more difficult to interpret in terms of tissue-specificity

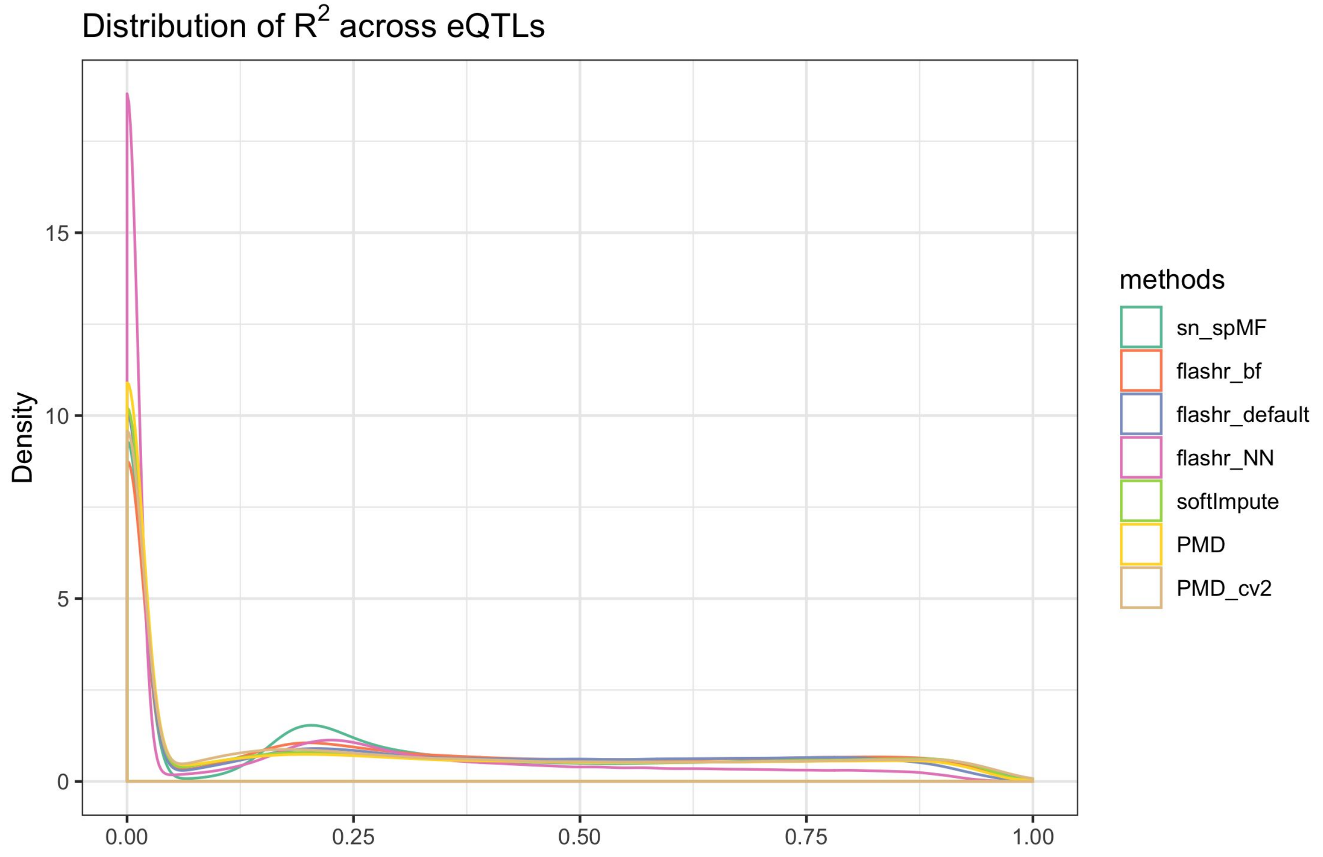

Figure S29. Distribution of R^2 for the tested eQTLs explained by factors assigned to them. For each eQTL, R^2 was computed as the variance of the weighted effect size explained by the variance of sum of weighted factors that have FDR < 0.05. Overall, sn_spMF and flashr_bf result in most eQTLs with R^2 > 0 (59% for sn_spMF, and 62% for flashr_bf). flashr_NN produced sparse, highly interpretable tissue factors, but did a poor job of fitting the actual eQTL effect sizes compared to flashr_default, flashr_bf or sn-spMF

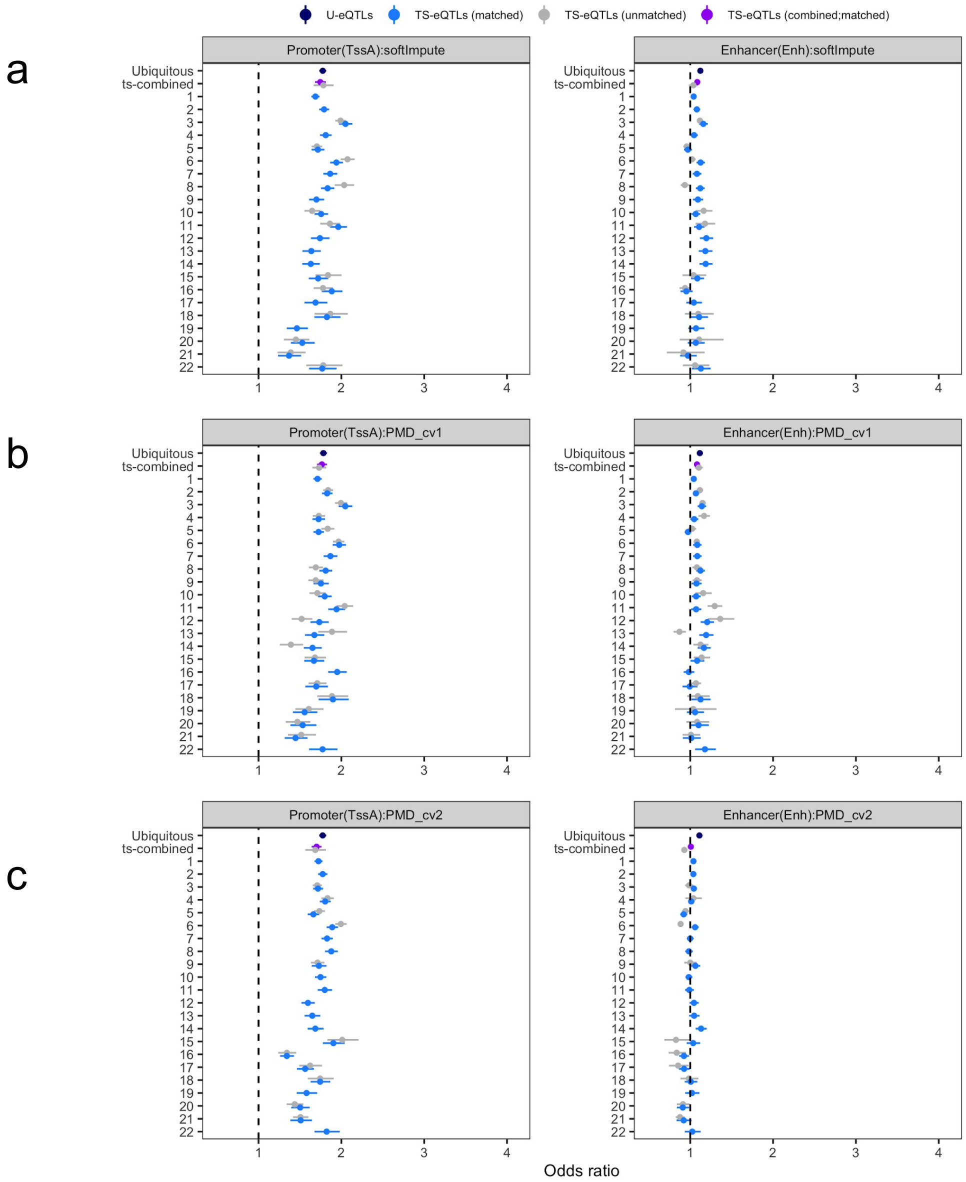

Figure S30. Enrichment analysis of cis-regulatory regions for u-eQTL variants and ts-eQTL variants from softImpute, PMD_cv1, and PMD_cv2. Y-axis represented eQTL types or the factor eQTLs are loaded on. X-axis represents odds ratio of enrichment relative to random SNPs matched for MAF and distance to gene TSS (see Methods) **(a)** For softImpute, both u-eQTL and ts-eQTL variants are both weakly enriched for enhancers, with ts-eQTLs not demonstrating greater enrichment than u-eQTLs (OR=1.1 and OR=1.1). **(b)** For PMD_cv1, u-eQTL and ts-eQTL variants are similarly weakly enriched for enhancers (OR=1.1 and OR=1.1). **(c)** For PMD_cv2, u-eQTL and ts-eQTL variants are again similarly weakly enriched for enhancers (OR=1.0 and OR=1.1).

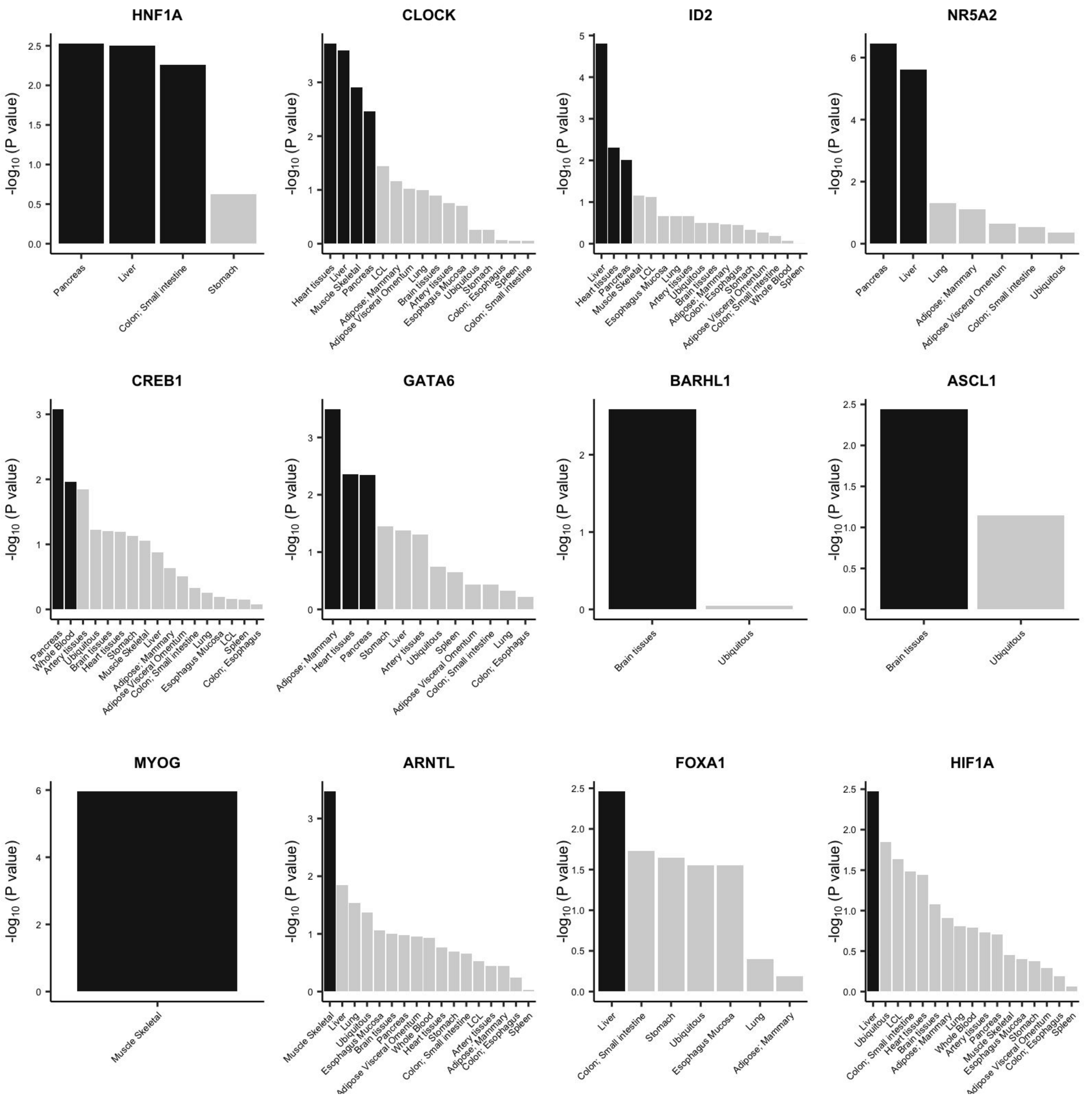

Figure S31. More examples of TFBS enrichment results from sn-spMF with known functions that are enriched in ts-eQTLs of the related tissues. In each panel, x-axis shows factors where the enrichment analysis for this TF were run, and y-axis shows the $-\log_{10}$(P value) from the enrichment analysis. Each bar in black represents that the TF is significantly enriched (FDR < 0.05) in the eQTLs loaded on the corresponding factor, and each bar in grey represents the un-significant enrichment tests (FDR >= 0.05).

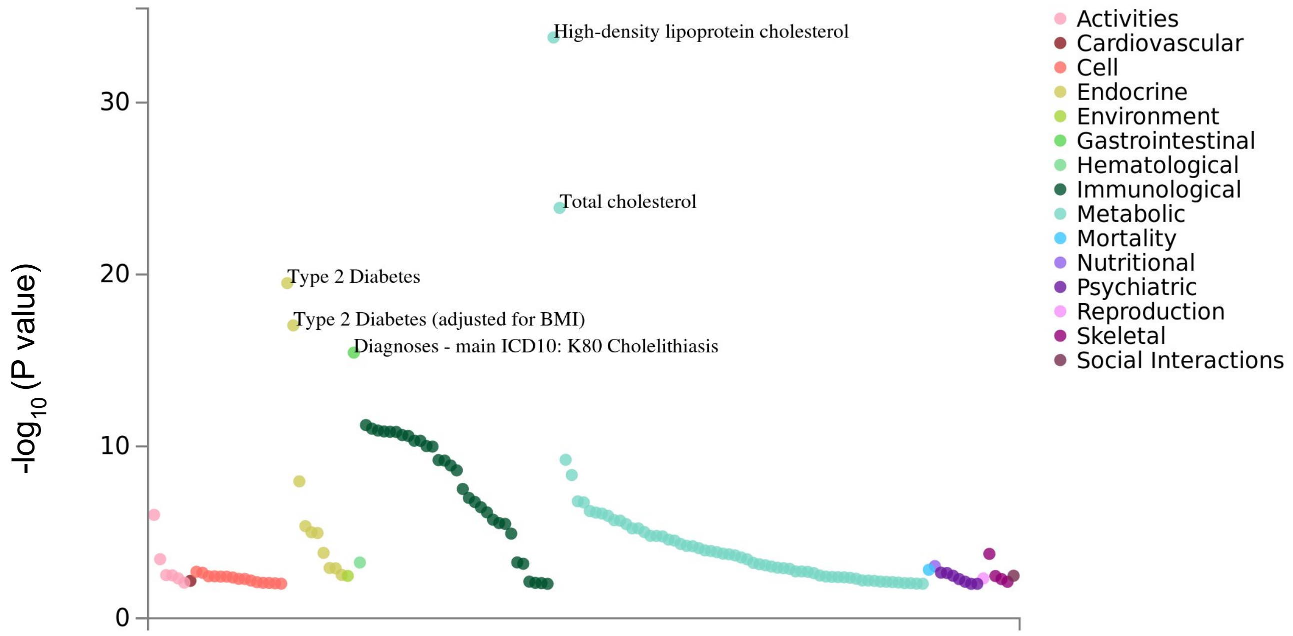

Figure S32. PheWAS plot that shows the GWAS results of the missense variant rs1800961 in HNF4A across multiple studies in the GWAS atlas (https://atlas.ctglab.nl/PheWAS). Each dot represents the GWAS result of rs1800961 in one study corresponding to one trait. The dots are ordered on the x-axis by trait domain and P-values.

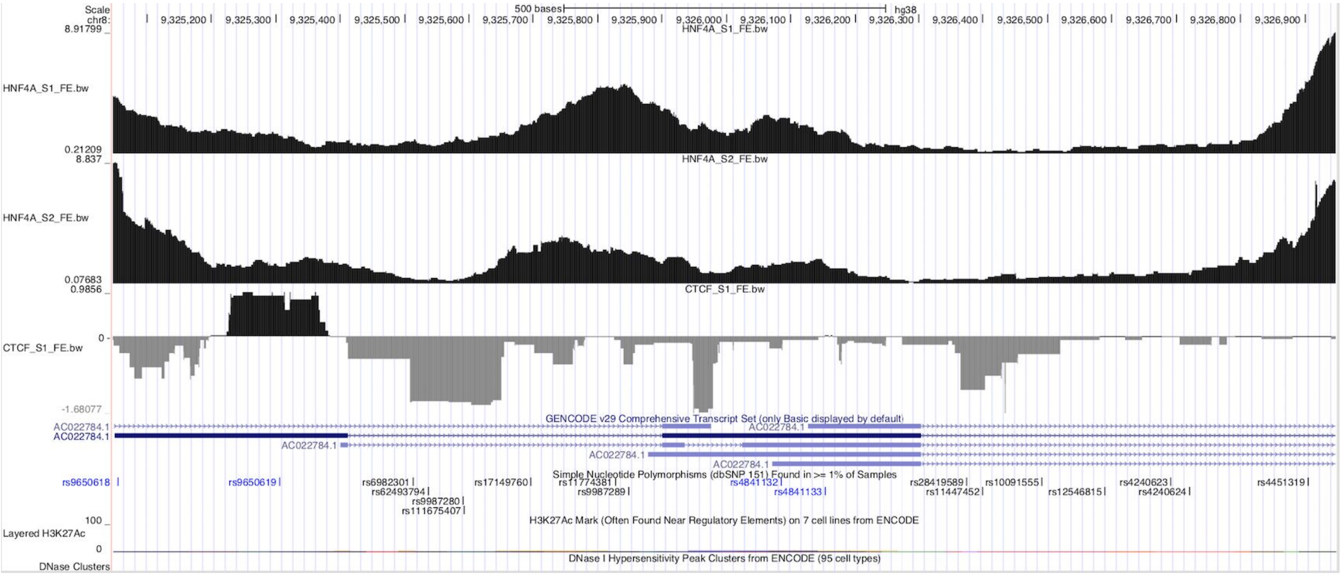

Figure S33. UCSC browser track showing binding signals of HNF4A and CTCF from ChIP-seq data on liver samples that was downloaded from ENCODE. The upper two rows of "HNF4A_S1_FE" and "HNF4A_S2_FE" show peak signals of HNF4A ChIP-seq data from two liver samples, using the corresponding control sample as background. The third row of "CTCF_S1_FE" shows peak sinal of CTCF using the corresponding control sample as the background. The plot shows that rs9987289 is in the peak region of HNF4A, but not in the peak region of CTCF.

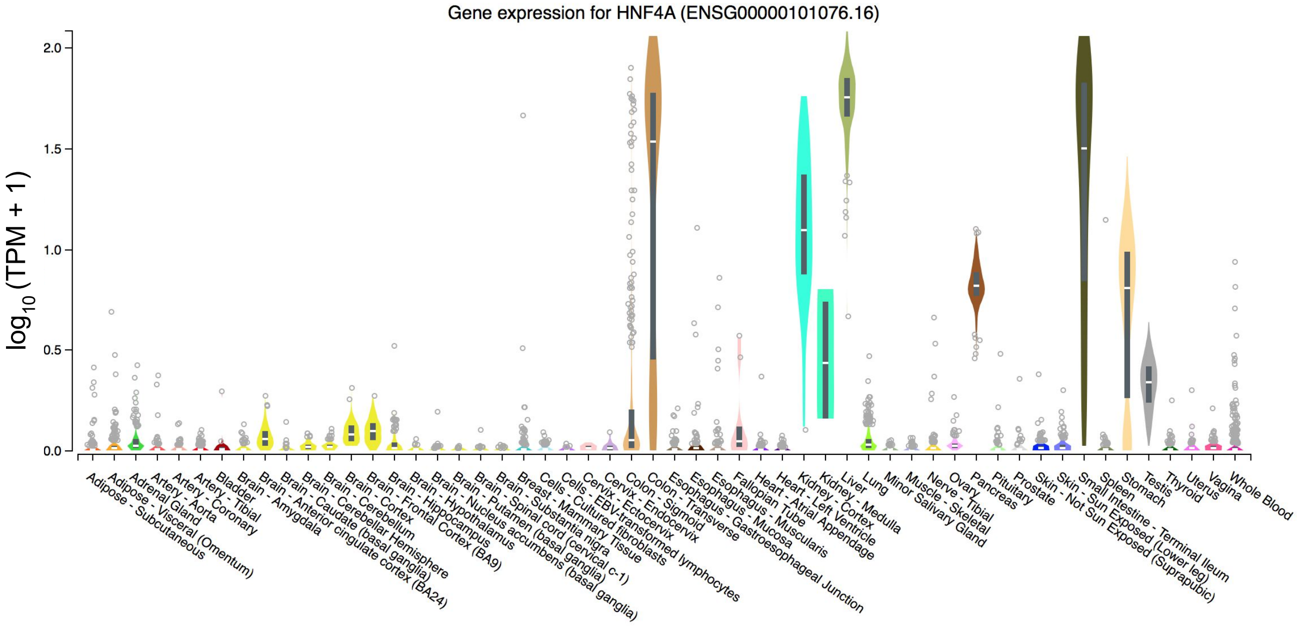

Figure S34. Expression level of HNF4A across tissues from GTEx v8. It shows that HNF4A have little expression (median TPM < 0.01 across samples) in LCL, muscle skeletal, heart left ventricle, brain hypothalamus, brain - substantia nigra, brain - nucleus accumbens (basal ganglia), brain - spinal cord (cervical c-1), brain - caudate (basal ganglia), brain - putamen (basal ganglia).

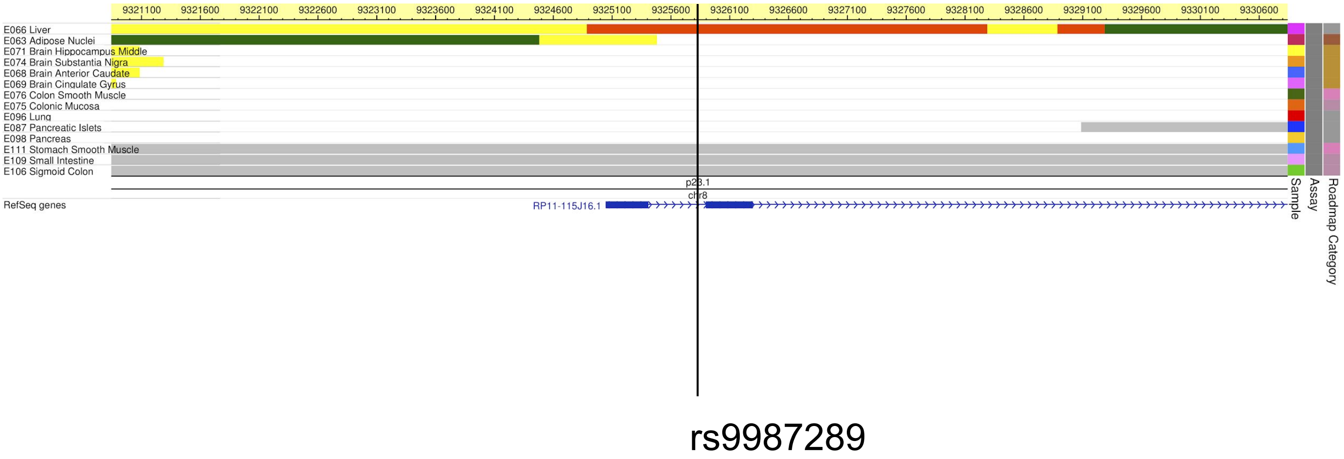

Figure S35. Prediction of chromatin states from chromHMM v1.10 for the chromosome region around rs9987289. For the filled colors: red - TssA (active TSS), yellow - Enh (enhancers), dark green - TxWk (weak transcription), white - Quies (Quiescent/Low). The plot was obtained from https://egg2.wustl.edu/roadmap/web_portal/.

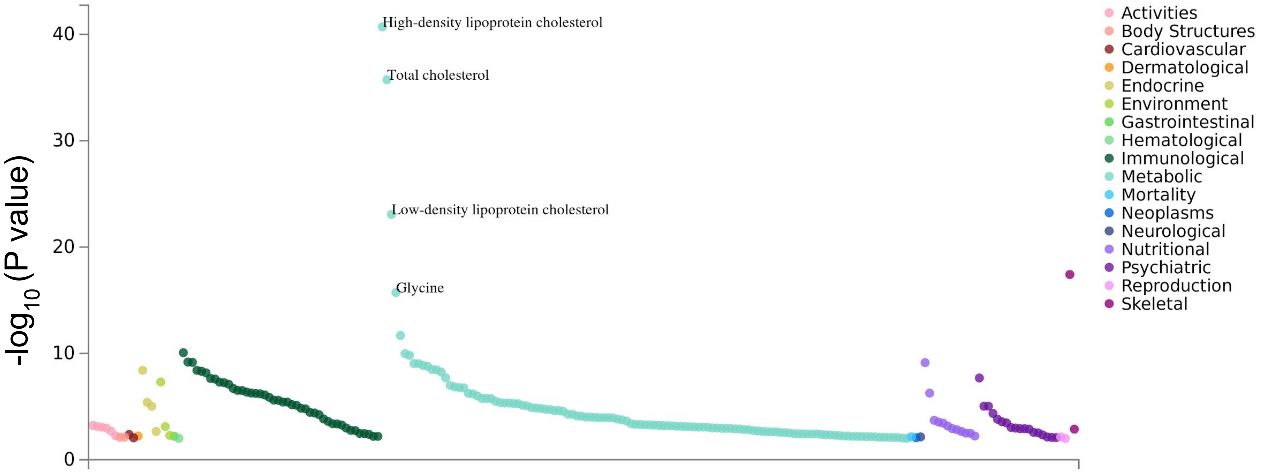

Figure S36. PheWAS plot that shows the GWAS results of the intron variant rs9987289 in HNF4A across multiple studies in the GWAS atlas (https://atlas.ctglab.nl/PheWAS). Each dot represents the GWAS result of rs9987289 in one study corresponding to one trait. The dots are ordered on the x-axis by trait domain and P-values.

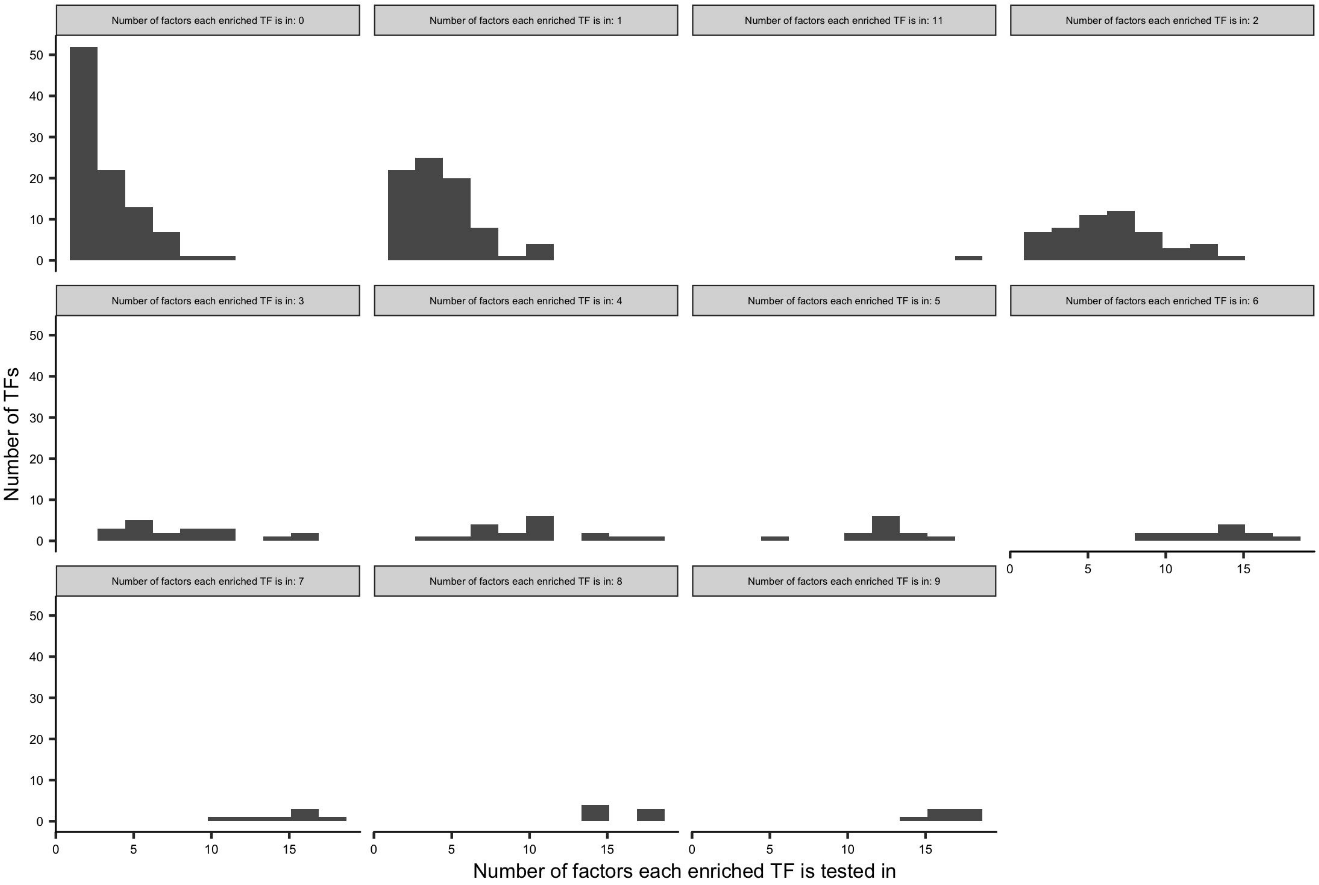

Figure S37. Each panel presents the TFs that are enriched in promoter regions for eQTLs in a specific number of factors. In each panel, the histogram shows the distribution of number of factors each TF is tested in.

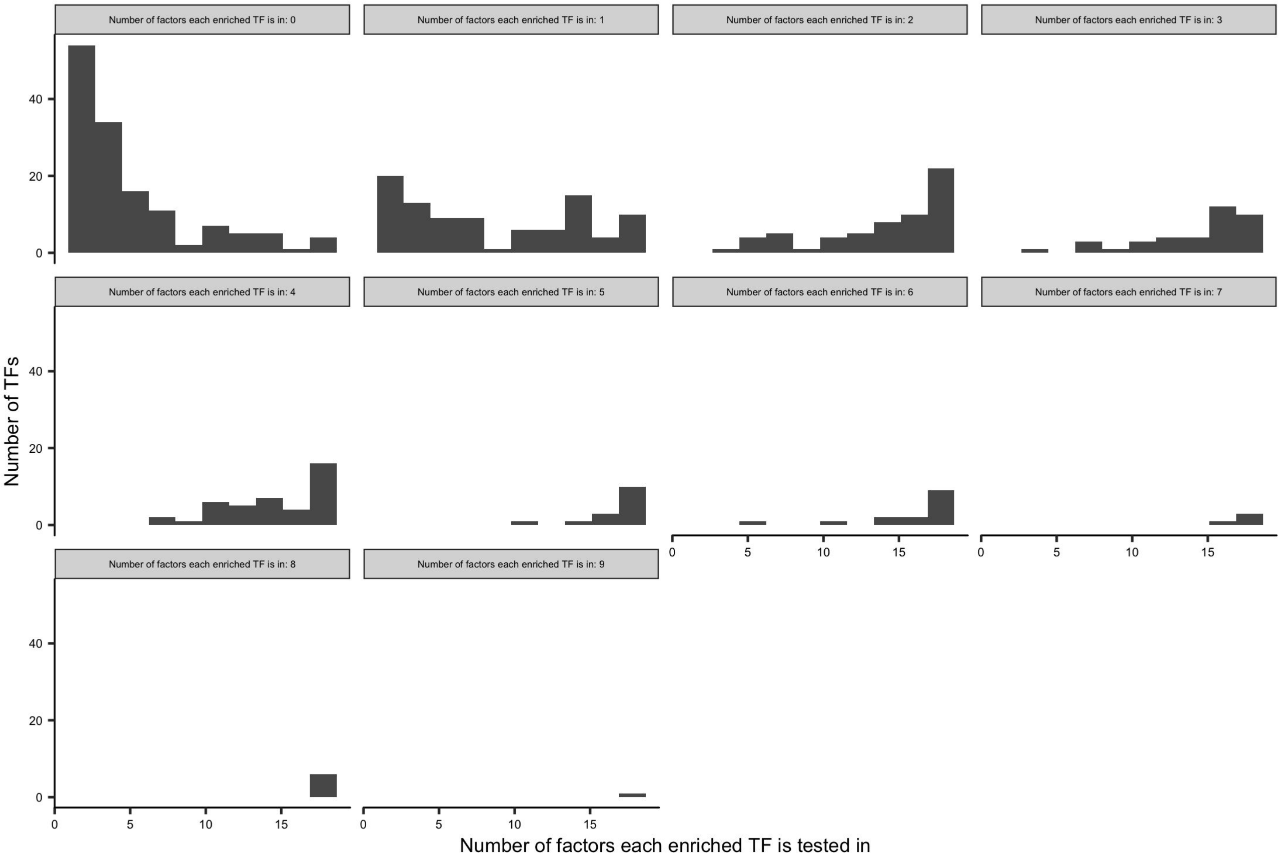

Figure S38. Each panel presents the TFs that are enriched in enhancer regions for eQTLs in a specific number of factors. In each panel, the histogram shows the distribution of number of factors each TF is tested in.
