# Supplementary material for: sn-spMF: matrix factorization informs tissue-specific genetic regulation of gene expression
Source: Genome Biol. 2020 Sep 11;21:235. doi: 10.1186/s13059-020-02129-6 (PMC7488540; doi:10.1186/s13059-020-02129-6)
Supplement: Supplementary file 2 — Additional file 2 Supplementary tables. Table S1 - S11. [file 13059_2020_2129_MOESM2_ESM.pdf]

Supplementary Table 1. Tissues not captured in tissue-specific factors by sn-spMF

**Tissue**	**Number of samples**
Adrenal gland	233
Cells cultured fibroblasts	483
Kidney cortex	73
Minor salivary gland	144
Ovary	167
Prostate	221
Uterus	129
Vagina	141

Supplementary Table 2. Number of u-eQTLs and ts-eQTLs captured by different methods

	**u-eQTLs**	**ts-eQTLs**
**sn_spMF**	1,076,761	76,976 - 431,585
**heuristic_1**	312,502	1,374 - 102,414
**heuristic_2**	175,637	1,460 - 201,584
**flashr_backfitting**	1,929,939	69,594 - 929,009
**flashr_default**	1,785,127	55,295 - 701,035
**flashr_NN**	243,467	54,306 - 338,615
**softImpute**	1,936,985	62,012 - 1,006,031
**PMD_cv1**	1,937,676	56,857 - 986,481
**PMD_cv2**	1,945,235	84,409 - 1,181,099

Explanation of table columns: **u-eQTLs**: number of eQTLs that load on the ubiquitous factor for each method. **ts-eQTLs:** number of eQTLs that load across tissue-specific factors for each method.

Supplementary Table 3. Subgroups of tissues used to define heuristic_2

Subgroup s	Tissues
1	49 tissues available in GTEx
2	Adipose Subcutaneous, Adipose Visceral Omentum
3	Adrenal Gland
4	Artery Aorta, Artery Coronary, Artery Tibial
5	Brain Amygdala, Brain Anterior cingulate cortex BA24, Brain Caudate basal ganglia, Brain Cerebellar Hemisphere, Brain Cerebellum, Brain Cortex, Brain Frontal Cortex BA9, Brain Hippocampus, Brain Hypothalamus, Brain Nucleus accumbens basal ganglia, Brain Putamen basal ganglia, Brain Spinal cord cervical c-1, Brain Substantia nigra
6	Cells EBV-transformed lymphocytes
7	Cells Cultured fibroblasts
8	Colon Sigmoid, Colon Transverse
9	Esophagus Gastroesophageal Junction, Esophagus Mucosa, Esophagus Muscularis
10	Heart Atrial Appendage, Heart Left Ventricle
11	Kidney Cortex
12	Liver
13	Lung
14	Minor Salivary Gland
15	Muscle Skeletal
16	Nerve Tibial
17	Ovary
18	Pancreas
19	Pituitary
20	Prostate
21	Skin Not Sun Exposed Suprapubic, Skin Sun Exposed Lower leg
22	Small Intestine Terminal Ileum
23	Spleen
24	Stomach

25	Testis
26	Thyroid
27	Uterus
28	Vagina
29	Whole Blood

Supplementary Table 4. Proportion of all tested eQTLs that have R^2 between model-predicted an actual effect sizes above a specific threshold:

	**$R^2 > 0$**	**$R^2 > 0.2$**	**$R^2 > 0.6$**
**sn_spMF**	59%	50%	21%
**flashr_backfitting**	62%	51%	22%
**flashr_default**	57%	47%	20%
**flashr_NN**	43%	34%	10%
**softImpute**	55%	44%	20%
**PMD_cv1**	55%	42%	19%
**PMD_cv2**	58%	45%	20%

Supplementary Table 5. Enrichment of u-eQTLs and ts-eQTLs in cis-regulatory regions

	**OR in promoter**		**OR in enhancer**	
	**u-eQTLs**	**ts-eQTLs**	**u-eQTLs**	**ts-eQTLs**
**sn_spMF**	1.9	1.5	1.0	1.3
**heuristic_1**	3.0	1.2	1.1	1.3
**heuristic_2**	3.1	1.2	1.1	1.4
**flashr_backfitting**	1.8	1.6	1.1	1.1
**flashr_default**	1.8	1.6	1.1	1.1
**flashr_NN**	2.1	1.5	0.9	1.1
**softImpute**	1.8	1.7	1.1	1.1

**PMD_cv1**	1.8	1.8	1.1	1.1
**PMD_cv2**	1.8	1.7	1.1	1.0

Supplementary Table 6. Number of enriched GO pathways for ts-eGenes

	**All ts-eGenes**	**Strictly defined ts-eGenes**
**sn_spMF**	546	45
**heuristic_1**	110	0
**heuristic_2**	421	1
**flashr_backfitting**	593	101
**flashr_default**	642	93
**flashr_NN**	453	7
**softImpute**	659	90
**PMD_cv1**	615	84
**PMD_cv2**	556	102

Supplementary Table 7. Number of enriched TFBS for u-eQTLs and ts-eQTLs in cis-regulatory regions

	**Promoter**		**Enhancer**	
	**u-eQTLs**	**ts-eQTLs**	**u-eQTLs**	**ts-eQTLs**
**sn_spMF**	136	181	39	264
**heuristic_1**	59	5	8	47
**heuristic_2**	97	9	4	54
**flashr_backfitting**	143	178	99	165
**flashr_default**	136	137	90	123
**flashr_NN**	70	104	2	109
**softImpute**	157	196	113	169
**PMD_cv1**	147	191	107	166
**PMD_cv2**	160	203	111	167

Supplementary Table 8. Enriched TFs with strong literature support

TF	Tissues	Reference (DOI)
BCL6	Adipose; Mammary	10.1073/pnas.1907308116
SREBF1	Adipose; Mammary	10.1038/srep00178
TWIST1	Adipose; Mammary	10.1016/j.cell.2009.01.051
SMAD4	Artery tissues	10.1128/MCB.00577-07
TWIST1	Artery tissues	10.1161/CIRCRESAHA.116.308870
LHX2	Brain tissues	10.1523/JNEUROSCI.3145-15.2016
OLIG1	Brain tissues	10.1038/nn.2600
SOX1	Brain tissues	10.1016/s0306-4522(03)00158-1
SOX2	Brain tissues	10.1242/dev.01204
SOX6	Brain tissues	10.1038/nn.2387
SOX9	Brain tissues	10.1523/JNEUROSCI.3199-16.2017
XBP1	Brain tissues	10.2119/molmed.2016.00229.
BCL6	Heart tissues	10.1016/S0008-6363(99)00007-3
CLOCK	Heart tissues	10.1161/hh1101.091190
FOXO1	Heart tissues	10.1093/cvr/cvs426
FOXP1	Heart tissues	10.1002/humu.22366
ID2	Heart tissues	10.1016/j.cell.2007.04.036
SOX6	Heart tissues	10.1073/pnas.97.8.4180
SOX9	Heart tissues	10.1073/pnas.0401711101
TWIST1	Heart tissues	10.1016/j.ydbio.2010.08.021
XBP1	Heart tissues	10.1111/acel.12460
CLOCK	Liver	10.1074/jbc.M304564200
FOXA1	Liver	10.1038/nature03649
FOXO1	Liver	10.1038/nm.2049
ID2	Liver	10.1074/jbc.M109.013961
MAFG	Liver	10.1016/j.cmet.2015.01.007
NFIL3	Liver	10.1016/j.metabol.2017.08.007

NR5A2	Liver	10.1016/j.ydbio.2016.07.019
XBP1	Liver	10.1074/jbc.M115.676239
CLOCK	Muscle Skeletal	10.1073/pnas.1014523107
FOXO1	Muscle Skeletal	10.18632/oncotarget.12891
PITX1	Muscle Skeletal	10.1016/j.ydbio.2006.06.055
SOX6	Muscle Skeletal	10.1002/dvdy.21223
SREBF1	Muscle Skeletal	10.1371/journal.pone.0050878
TEAD1	Muscle Skeletal	10.1074/jbc.M113.515817
ATF4	Pancreas	10.1016/j.cmet.2008.01.008
CLOCK	Pancreas	10.1038/nature09253
FOXO1	Pancreas	10.1210/en.2015-1852
FOXP1	Pancreas	10.1007/s00125-015-3635-3
ID2	Pancreas	10.1007/s12020-008-9039-0
NEUROD1	Pancreas	10.1101/gad.9.8.1009
NKX6-1	Pancreas	10.1016/j.celrep.2013.08.010
NR5A2	Pancreas	10.1016/j.ydbio.2016.07.019
SOX6	Pancreas	10.1074/jbc.M700460200
SREBF1	Pancreas	10.1194/jlr.M700533-JLR200
TEAD1	Pancreas	10.1038/ncb3160
XBP1	Pancreas	10.1038/sj.emboj.7600903
FLI1	Whole Blood	10.1016/j.cub.2008.07.048
FOXO1	Whole Blood	10.1038/ncomms11023
NFIL3	Whole Blood	10.1136/annrheumdis-2018-213764
RUNX1	Whole Blood	10.1038/emboj.2012.275

Supplementary Table 9. Samples from Roadmap Epigenomics project mapped to GTEx tissues

Roadmap sample ID	Roadmap Tissue	GTEx Tissues
E063	Adipose Nuclei	Adipose Subcutaneous, Adipose Visceral Omentum
E066	Adult Liver	Liver
E065	Aorta	Artery Aorta
E067	Brain Angular Gyrus	Brain Cortex
E068	Brain Anterior Caudate	Brain Caudate basal ganglia
E069	Brain Cingulate Gyrus	Brain Anterior cingulate cortex BA24
E071	Brain Hippocampus Middle	Brain Hippocampus
E072	Brain Inferior Temporal Lobe	Brain Cortex, Brain Frontal Cortex BA9
E073	Brain Mid Frontal Lobe	Brain Cortex, Brain Frontal Cortex BA9
E027	Breast Myoepithelial Cells	Breast Mammary Tissue
E075	Colonic Mucosa	Colon Transverse
E076	Colon Smooth Muscle	Colon Transverse
E106	Sigmoid Colon	Colon Sigmoid
E079	Esophagus	Esophagus Gastroesophageal Junction, Esophagus Mucosa, Esophagus Muscularis
E116	GM12878 Lymphoblastoid	Cells EBV-transformed lymphocytes
E095	Left Ventricle	Heart Left Ventricle
E096	Lung	Lung
E097	Ovary	Ovary
E098	Pancreas	Pancreas
E062	Peripheral Blood Mononuclear Primary Cells	Whole Blood
E104	Right Atrium	Heart Atrial Appendage
E108	Skeletal Muscle Female	Muscle Skeletal
E107	Skeletal Muscle Male	Muscle Skeletal
E109	Small Intestine	Small Intestine Terminal Ileum
E113	Spleen	Spleen
E110	Stomach Mucosa	Stomach
E111	Stomach Smooth Muscle	Stomach

Supplementary Table 10. DNase-seq data from ENCODE project

ENCODE Accession	ENCODE sample tissue	GTEx tissue
ENCFF958GWR, ENCFF954PTR	Omental fat pad	Adipose Subcutaneous, Adipose Visceral Omentum
ENCFF042VKK, ENCFF085NOG, ENCFF108XQG, ENCFF217MXO, ENCFF315CSH, ENCFF367BEU, ENCFF587SIS, ENCFF675UKK, ENCFF688ZWO, ENCFF896DOA, ENCFF977OWF	Adrenal gland	Adrenal Gland
ENCFF968IAI	Ascending aorta	Artery Aorta
ENCFF822UQG, ENCFF178BNR	Coronary artery	Artery Coronary
ENCFF048ZGK, ENCFF267DGC	Tibial artery	Artery Tibial
ENCFF240ECT	Caudate nucleus	Brain Caudate basal ganglia
ENCFF053XFC, ENCFF337NAS	Cerebellar cortex	Brain Cerebellar Hemisphere
ENCFF732MQW, ENCFF966DRW	Cerebellum	Brain Cerebellum
ENCFF255NTQ, ENCFF611EHQ, ENCFF631HBT, ENCFF855HES	Frontal cortex	Brain Frontal Cortex BA9
ENCFF026XWM	Putamen	Brain Putamen basal ganglia
ENCFF421NEH, ENCFF469JHU	Sigmoid colon	Colon Sigmoid
ENCFF134KRY, ENCFF159SOA, ENCFF161NFM, ENCFF384WXP, ENCFF791HOY,	Transverse colon	Colon Transverse
ENCFF146AEB	Esophagus muscularis mucosa	Esophagus Gastroesophageal Junction, Esophagus Mucosa, Esophagus Muscularis
ENCFF146VYU, ENCFF778BRJ, ENCFF794SOC, ENCFF855YGO	Heart left ventricle	Heart Atrial Appendage, Heart Left Ventricle
ENCFF172XNI	Left cardiac atrium	

ENCFF207NXB, ENCFF289IMF	Heart right ventricle	
ENCFF036JUB, ENCFF153WQN, ENCFF183AEI, ENCFF262FHU, ENCFF270GNM, ENCFF305WVB, ENCFF402THI, ENCFF484XHW, ENCFF578BEO, ENCFF765BJR, ENCFF812GJU, ENCFF845VOI, ENCFF871EKB, ENCFF916NTG, ENCFF932ATD	Kidney	Kidney Cortex
ENCFF081JVT, ENCFF468NND, ENCFF512TSJ	Liver	Liver
ENCFF475HWF	Right lobe of liver	
ENCFF318TOW, ENCFF353SVP, ENCFF439ZRL, ENCFF484YOE, ENCFF588WQL, ENCFF601TZC, ENCFF642HTL, ENCFF671CWO, ENCFF676GRC, ENCFF690UKD, ENCFF796EIB, ENCFF929FIK, ENCFF944FSO	Left lung	Lung
ENCFF449XXS, ENCFF486CWL, ENCFF791OUO	Upper lobe of left lung	
ENCFF024SOP, ENCFF115HTH, ENCFF148PHO, ENCFF348CJE, ENCFF363XQF, ENCFF395KUT, ENCFF422YFH, ENCFF679QGU, ENCFF811RTH, ENCFF889NTH, ENCFF909JGU, ENCFF913NRZ, ENCFF962JWU, ENCFF978OUM, ENCFF992VNB	Lung	
ENCFF157KGS, ENCFF277WMS, ENCFF281HKU, ENCFF352RNR, ENCFF516FXF, ENCFF586UYY, ENCFF604AQG, ENCFF628MPB, ENCFF785ORF, ENCFF796WDQ, ENCFF941EJJ	Right lung	
ENCFF028CVN, ENCFF036PYG, ENCFF040WPR, ENCFF041NEG, ENCFF213IAV, ENCFF229KSL, ENCFF246TUN, ENCFF262NZB, ENCFF308QRZ, ENCFF334ENU, ENCFF349CIP, ENCFF475YRW, ENCFF615LEO, ENCFF755PMB, ENCFF771EKC, ENCFF886DDL, ENCFF994YDK	Muscle of arm	Muscle Skeletal

ENCFF016PCJ, ENCFF062LJL, ENCFF182YXK, ENCFF191MBC, ENCFF365RKF, ENCFF376WVL, ENCFF417IJL, ENCFF433VTN, ENCFF468TOZ, ENCFF735DNU, ENCFF758REB, ENCFF766PCO, ENCFF831RQP, ENCFF897TZA, ENCFF994ALS	Muscle of back	
ENCFF011UBS, ENCFF031RMC, ENCFF058UNN, ENCFF067VHZ, ENCFF138LZU, ENCFF175BCP, ENCFF241QSX, ENCFF283UJD, ENCFF470XXM, ENCFF699XQF, ENCFF874GGX, ENCFF896ZUQ, ENCFF970QZI	Muscle of leg	
ENCFF022UVJ, ENCFF383OJO, ENCFF443ZJX	Muscle of trunk	
ENCFF614SOO, ENCFF643WVI	Tibial nerve	Nerve Tibial
ENCFF111IXG, ENCFF342PZX, ENCFF701ZFB, ENCFF936ENC	Ovary	Ovary
ENCFF398ENA, ENCFF535LEW	Pancreas	Pancreas
ENCFF569WWH, ENCFF627DYO, ENCFF779JBV, ENCFF963BGI	Body of pancreas	
ENCFF228ZTQ	Lower leg skin	Skin Not Sun Exposed Suprapubic,<br>Skin Sun Exposed Lower leg
ENCFF019PSW, ENCFF087XDG, ENCFF130CZB, ENCFF274NTF, ENCFF333MTL, ENCFF412ATV, ENCFF424PWV, ENCFF571UWP, ENCFF617TGM, ENCFF720DUQ, ENCFF731WZI, ENCFF758VXS, ENCFF885IBS	Small intestine	Small Intestine Terminal Ileum
ENCFF376YIY, ENCFF534XLO	Spleen	Spleen
ENCFF009YJE, ENCFF024HZS, ENCFF187BOF, ENCFF227HYU, ENCFF272WHU, ENCFF278ROU, ENCFF376EBF, ENCFF457SNJ, ENCFF523INF, ENCFF556WLI, ENCFF631XSP, ENCFF694LGV, ENCFF709TJW, ENCFF716YVE,	Stomach	Stomach

ENCFF749DUT, ENCFF751PUA, ENCFF765AZQ, ENCFF785AIA, ENCFF885VHR, ENCFF967LJZ, ENCFF988ZPF		
ENCFF018TWY, ENCFF102TYW, ENCFF618EIJ	Testis	Testis
ENCFF440OAH, ENCFF460WME, ENCFF652DKF, ENCFF856OWH	Thyroid gland	Thyroid
ENCFF514GYQ	Uterus	Uterus
ENCFF018IDK, ENCFF329QLI	Vagina	Vagina
ENCFF808CMV, ENCFF736PAW, ENCFF209WIT, ENCFF359WHG , ENCFF131SCF, ENCFF126JXB, ENCFF138YRO, ENCFF668TER, ENCFF839YTP, ENCFF835SMB, ENCFF410MHQ, ENCFF046KTX	T cell	Whole Blood
ENCFF698QNG, ENCFF497QQN, ENCFF829UTZ, ENCFF355LDD, ENCFF238LUI, ENCFF541VKE, ENCFF841XRI, ENCFF507JIF, ENCFF444ZRC, ENCFF805PHJ	B cell	
ENCFF860NPB, ENCFF007TSW, ENCFF335JED, ENCFF438EZP, ENCFF228QBR, ENCFF915XQC, ENCFF154TFX, ENCFF859JIA	CD14 positive monocytes	

Supplementary Table 11. ChIP-seq data from ENCODE

TF	Experiment
HNF4A	ENCSR445QRF, ENCSR601OGE
CTCF	ENCSR254YRM
