# Supplementary material for: sn-spMF: matrix factorization informs tissue-specific genetic regulation of gene expression
Source: Genome Biol. 2020 Sep 11;21:235. doi: 10.1186/s13059-020-02129-6 (PMC7488540; doi:10.1186/s13059-020-02129-6)
Supplement: Supplementary file 3 — Additional file 3 GTEx Consortium Information. [file 13059_2020_2129_MOESM3_ESM.pdf]

Authors

GTEx Consortium*

Laboratory and Data Analysis Coordinating Center (LDACC): François Aguet[1], Shankara Anand[1], Kristin G Ardlie[1], Stacey Gabriel[1], Gad A Getz[1,2,3], Aaron Graubert[1], Kane Hadley[1], Robert E Handsaker[4,5,6], Katherine H Huang[1], Seva Kashin[4,5,6], Xiao Li[1], Daniel G MacArthur[5,7], Samuel R Meier[1], Jared L Nedzel[1], Duyen T Nguyen[1], Ayellet V Segrè[1,8], Ellen Todres[1]

Analysis Working Group (funded by GTEx project grants):
François Aguet[1], Shankara Anand[1], Kristin G Ardlie[1], Brunilda Balliu[9], Alvaro N Barbeira[10], Alexis Battle[11,12], Rodrigo Bonazzola[10], Andrew Brown[13,14], Christopher D Brown[15], Stephane E Castel[16,17], Donald F Conrad[18,19], Daniel J Cotter[20], Nancy Cox[21], Sayantan Das[22], Olivia M de Goede[20], Emmanouil T Dermitzakis[13,23,24], Jonah Einson[25,16], Barbara E Engelhardt[26,27], Eleazar Eskin[28], Tiffany Y Eulalio[29], Nicole M Ferraro[29], Elise D Flynn[16,17], Laure Fresard[30], Eric R Gamazon[31,32,33,21], Diego Garrido-Martín[34], Nicole R Gay[20], Gad A Getz[1,2,3], Michael J Gloudemans[29], Aaron Graubert[1], Roderic Guigó[34,35], Kane Hadley[1], Andrew R Hame[18,1], Robert E Handsaker[4,5,6], Yuan He[11], Paul J Hoffman[16], Farhad Hormozdiari[36,1], Lei Hou[37,1], Katherine H Huang[1], Hae Kyung Im[10], Brian Jo[26,27], Silva Kasela[16,17], Seva Kashin[4,5,6], Manolis Kellis[37,1], Sarah Kim-Hellmuth[16,17,38], Alan Kwong[22], Tuuli Lappalainen[16,17], Xiao Li[1], Xin Li[30], Yanyu Liang[10], Daniel G MacArthur[5,7], Serghei Mangul[28,39], Samuel R Meier[1], Pejman Mohammadi[16,17,40,41], Stephen B Montgomery[30,20], Manuel Muñoz-Aguirre[34,42], Daniel C Nachun[30], Jared L Nedzel[1], Duyen T Nguyen[1], Andrew B Nobel[43], Meritxell Oliva[10,44], YoSon Park[15,45], Yongjin Park[37,1], Princy Parsana[12], Abhiram S Rao[46], Ferran Reverter[47], John M Rouhana[8,1], Chiara Sabatti[48], Ashis Saha[12], Ayellet V Segrè[1,8,] Andrew D Skol[10,49], Matthew Stephens[50], Barbara E Stranger[10,51], Benjamin J Strober[11], Nicole A Teran[30], Ellen Todres[1], Ana Viñuela[52,13,23,24], Gao Wang[50], Xiaoquan Wen[22], Fred Wright[53], Valentin Wucher[34], Yuxin Zou[54]

Analysis Working Group (not funded by GTEx project grants): Pedro G Ferreira[55,56,57,58], Gen Li[59], Marta Melé[60], Esti Yeger-Lotem[61,62]

Leidos Biomedical - Project Management: Mary E Barcus[63], Debra Bradbury[63], Tanya Krubit[63], Jeffrey A McLean[63], Liqun Qi[63], Karna Robinson[63], Nancy V Roche[63], Anna M Smith[63], Leslie Sobin[63], David E Tabor[63], Anita Undale[63]

Biospecimen collection source sites: Jason Bridge[64], Lori E Brigham[65], Barbara A Foster[66], Bryan M Gillard[66], Richard Hasz[67], Marcus Hunter[68], Christopher Johns[69], Mark Johnson[70], Ellen Karasik[66], Gene Kopen[71], William F Leinweber[71], Alisa McDonald[71], Michael T Moser[66], Kevin Myer[68], Kimberley D Ramsey[66], Brian Roe[68], Saboor Shad[71], Jeffrey A Thomas[71,70], Gary Walters[70], Michael Washington[70], Joseph Wheeler[69]

Biospecimen core resource: Scott D Jewell[72,] Daniel C Rohrer[72], Dana R Valley[72]

Brain bank repository: David A Davis[73], Deborah C Mash[73]

Pathology: Mary E Barcus[63], Philip A Branton[74], Leslie Sobin[63]

ELSI study: Laura K Barker[75], Heather M Gardiner[75], Maghboeba Mosavel[76], Laura A Siminoff[75]
Genome Browser Data Integration & Visualization: Paul Flicek[77], Maximilian Haeussler[78], Thomas Juettemann[77], W James Kent[78], Christopher M Lee[78], Conner C Powell[78], Kate R Rosenbloom[78], Magali Ruffier[77], Dan Sheppard[77], Kieron Taylor[77], Stephen J Trevanion[77], Daniel R Zerbino[77]
eGTEx groups: Nathan S Abell[20], Joshua Akey[79], Lin Chen[44], Kathryn Demanelis[44], Jennifer A Doherty[80], Andrew P Feinberg[81], Kasper D Hansen[82], Peter F Hickey[83], Lei Hou[37,1], Farzana Jasmine[44], Lihua Jiang[20], Rajinder Kaul[84,85], Manolis Kellis[37,1], Muhammad G Kibriya[44], Jin Billy Li[20], Qin Li[20], Shin Lin[86], Sandra E Linder[20], Stephen B Montgomery[30,20], Meritxell Oliva[10,44], Yongjin Park[37,1], Brandon L Pierce[44], Lindsay F Rizzardi[87], Andrew D Skol[10,49], Kevin S Smith[30], Michael Snyder[20], John Stamatoyannopoulos[84,88], Barbara E Stranger[10,51], Hua Tang[20], Meng Wang[20]
NIH program management: Philip A Branton[74], Latarsha J Carithers[74,89], Ping Guan[74], Susan E Koester[90], A. Roger Little[91], Helen M Moore[74], Concepcion R Nierras[92], Abhi K Rao[74], Jimmie B Vaught[74], Simona Volpi[93]

Affiliations

1. The Broad Institute of MIT and Harvard, Cambridge, MA, USA
2. Cancer Center and Department of Pathology, Massachusetts General Hospital, Boston, MA, USA
3. Harvard Medical School, Boston, MA, USA
4. Department of Genetics, Harvard Medical School, Boston, MA, USA
5. Program in Medical and Population Genetics, The Broad Institute of Massachusetts Institute of Technology and Harvard University, Cambridge, MA, USA
6. Stanley Center for Psychiatric Research, Broad Institute, Cambridge, MA, USA
7. Analytic and Translational Genetics Unit, Massachusetts General Hospital, Boston, MA, USA
8. Ocular Genomics Institute, Massachusetts Eye and Ear, Harvard Medical School, Boston, MA, USA
9. Department of Biomathematics, University of California, Los Angeles, Los Angeles, CA, USA
10. Section of Genetic Medicine, Department of Medicine, The University of Chicago, Chicago, IL, USA
11. Department of Biomedical Engineering, Johns Hopkins University, Baltimore, MD, USA
12. Department of Computer Science, Johns Hopkins University, Baltimore, MD, USA
13. Department of Genetic Medicine and Development, University of Geneva Medical School, Geneva, Switzerland
14. Population Health and Genomics, University of Dundee, Dundee, Scotland, UK
15. Department of Genetics, University of Pennsylvania, Perelman School of Medicine, Philadelphia, PA, USA
16. New York Genome Center, New York, NY, USA
17. Department of Systems Biology, Columbia University, New York, NY, USA
18. Department of Genetics, Washington University School of Medicine, St. Louis, Missouri, USA
19. Division of Genetics, Oregon National Primate Research Center, Oregon Health & Science University, Portland, OR, USA
20. Department of Genetics, Stanford University, Stanford, CA, USA
21. Division of Genetic Medicine, Department of Medicine, Vanderbilt University Medical Center, Nashville, TN, USA

22. Department of Biostatistics, University of Michigan, Ann Arbor, MI, USA
23. Institute for Genetics and Genomics in Geneva (iGE3), University of Geneva, Geneva, Switzerland
24. Swiss Institute of Bioinformatics, Geneva, Switzerland
25. Department of Biomedical Informatics, Columbia University, New York, NY, USA
26. Department of Computer Science, Princeton University, Princeton, NJ, USA
27. Center for Statistics and Machine Learning, Princeton University, Princeton, NJ, USA
28. Department of Computer Science, University of California, Los Angeles, Los Angeles, CA, USA
29. Program in Biomedical Informatics, Stanford University School of Medicine, Stanford, CA, USA
30. Department of Pathology, Stanford University, Stanford, CA, USA
31. Data Science Institute, Vanderbilt University, Nashville, TN, USA
32. Clare Hall, University of Cambridge, Cambridge, UK
33. MRC Epidemiology Unit, University of Cambridge, Cambridge, UK
34. Centre for Genomic Regulation (CRG), The Barcelona Institute for Science and Technology, Barcelona, Catalonia, Spain
35. Universitat Pompeu Fabra (UPF), Barcelona, Catalonia, Spain
36. Department of Epidemiology, Harvard T.H. Chan School of Public Health, Boston, MA, USA
37. Computer Science and Artificial Intelligence Laboratory, Massachusetts Institute of Technology, Cambridge, MA, USA
38. Statistical Genetics, Max Planck Institute of Psychiatry, Munich, Germany
39. Department of Clinical Pharmacy, School of Pharmacy, University of Southern California, Los Angeles, CA, USA
40. Scripps Research Translational Institute, La Jolla, CA, USA
41. Department of Integrative Structural and Computational Biology, The Scripps Research Institute, La Jolla, CA, USA
42. Department of Statistics and Operations Research, Universitat Politècnica de Catalunya (UPC), Barcelona, Catalonia, Spain
43. Department of Statistics and Operations Research and Department of Biostatistics, University of North Carolina, Chapel Hill, NC, USA
44. Department of Public Health Sciences, The University of Chicago, Chicago, IL, USA
45. Department of Systems Pharmacology and Translational Therapeutics, University of Pennsylvania, Perelman School of Medicine, Philadelphia, PA, USA
46. Department of Bioengineering, Stanford University, Stanford, CA, USA
47. Department of Genetics, Microbiology and Statistics, University of Barcelona, Barcelona. Spain.
48. Departments of Biomedical Data Science and Statistics, Stanford University, Stanford, CA, USA
49. Department of Pathology and Laboratory Medicine, Ann & Robert H. Lurie Children's Hospital of Chicago, Chicago, IL, USA
50. Department of Human Genetics, University of Chicago, Chicago, IL, USA
51. Center for Genetic Medicine, Department of Pharmacology, Northwestern University, Feinberg School of Medicine, Chicago, IL, USA
52. Department of Twin Research and Genetic Epidemiology, King's College London, London, UK
53. Bioinformatics Research Center and Departments of Statistics and Biological Sciences, North Carolina State University, Raleigh, NC, USA
54. Department of Statistics, University of Chicago, Chicago, IL, USA
55. Department of Computer Sciences, Faculty of Sciences, University of Porto, Porto, Portugal
56. Instituto de Investigação e Inovação em Saúde, University of Porto, Porto, Portugal
57. Institute of Molecular Pathology and Immunology, University of Porto, Porto, Portugal
58. Laboratory of Artificial Intelligence and Decision Support, Institute for Systems and Computer Engineering, Technology and Science, Porto, Portugal
59. Columbia University Mailman School of Public Health, New York, NY, USA
60. Life Sciences Department, Barcelona Supercomputing Center, Barcelona, Spain

61. Department of Clinical Biochemistry and Pharmacology, Ben-Gurion University of the Negev, Beer-Sheva, Israel
62. National Institute for Biotechnology in the Negev, Beer-Sheva, Israel
63. Leidos Biomedical, Rockville, MD, USA
64. UNYTS, Buffalo, NY, USA
65. Washington Regional Transplant Community, Annandale, VA, USA
66. Therapeutics, Roswell Park Comprehensive Cancer Center, Buffalo, NY, USA
67. Gift of Life Donor Program, Philadelphia, PA, USA
68. LifeGift, Houston, TX, USA
69. Center for Organ Recovery and Education, Pittsburgh, PA, USA
70. LifeNet Health, Virginia Beach, VA. USA
71. National Disease Research Interchange, Philadelphia, PA, USA
72. Van Andel Research Institute, Grand Rapids, MI, USA
73. Department of Neurology, University of Miami Miller School of Medicine, Miami, FL, USA
74. Biorepositories and Biospecimen Research Branch, Division of Cancer Treatment and Diagnosis, National Cancer Institute, Bethesda, MD, USA
75. Temple University, Philadelphia, PA, USA
76. Virginia Commonwealth University, Richmond, VA, USA
77. European Molecular Biology Laboratory, European Bioinformatics Institute, Hinxton, United Kingdom
78. Genomics Institute, University of California Santa Cruz, Santa Cruz, CA, USA
79. Carl Icahn Laboratory, Princeton University, Princeton, NJ, USA
80. Department of Population Health Sciences, The University of Utah, Salt Lake City, Utah, USA
81. Departments of Medicine, Biomedical Engineering, and Mental Health, Johns Hopkins University, Baltimore, MD, USA
82. Department of Biostatistics, Bloomberg School of Public Health, Johns Hopkins University, Baltimore, MD, USA
83. Department of Medical Biology, The Walter and Eliza Hall Institute of Medical Research, Parkville, Victoria, Australia
84. Altius Institute for Biomedical Sciences, Seattle, WA, USA
85. Division of Genetics, University of Washington, Seattle, WA, University of Washington, Seattle, WA, USA
86. Department of Cardiology, University of Washington, Seattle, WA, USA
87. HudsonAlpha Institute for Biotechnology, Huntsville, AL, USA
88. Genome Sciences, University of Washington, Seattle, WA, USA
89. National Institute of Dental and Craniofacial Research, Bethesda, MD, USA
90. Division of Neuroscience and Basic Behavioral Science, National Institute of Mental Health, National Institutes of Health, Bethesda, MD, USA
91. National Institute on Drug Abuse, Bethesda, MD, USA
92. Office of Strategic Coordination, Division of Program Coordination, Planning and Strategic Initiatives, Office of the Director, National Institutes of Health, Rockville, MD, USA
93. Division of Genomic Medicine, National Human Genome Research Institute, Bethesda, MD, USA

Funding

This work was supported by the Common Fund of the Office of the Director, U.S. National Institutes of Health, and by NCI, NHGRI, NHLBI, NIDA, NIMH, NIA, NIAID, and NINDS through NIH contracts HHSN261200800001E (Leidos Prime contract with NCI: A.M.S., D.E.T., N.V.R., J.A.M., L.S., M.E.B., L.Q., T.K., D.B., K.R., A.U.), 10XS170 (NDRI: W.F.L., J.A.T., G.K., A.M., S.S., R.H., G.Wa., M.J., M.Wa., L.E.B., C.J., J.W., B.R., M.Hu., K.M., L.A.S.,

H.M.G., M.Mo., L.K.B.), 10XS171 (Roswell Park Cancer Institute: B.A.F., M.T.M., E.K., B.M.G., K.D.R., J.B.), 10X172 (Science Care Inc.), 12ST1039 (IDOX), 10ST1035 (Van Andel Institute: S.D.J., D.C.R., D.R.V.), HHSN268201000029C (Broad Institute: F.A., G.G., K.G.A., A.V.S., X.Li., E.T., S.G., A.G., S.A., K.H.H., D.T.N., K.H., S.R.M., J.L.N.), 5U41HG009494 (F.A., G.G., K.G.A.), and through NIH grants R01 DA006227-17 (Univ. of Miami Brain Bank: D.C.M., D.A.D.), Supplement to University of Miami grant DA006227 (D.C.M., D.A.D.), R01 MH090941 (Univ. of Geneva), R01 MH090951 and R01 MH090937 (Univ. of Chicago), R01 MH090936 (Univ. of North Carolina–Chapel Hill), R01MH101814 (M.M-A., V.W., S.B.M., R.G., E.T.D., D.G-M., A.V.), U01HG007593 (S.B.M.), R01MH101822 (C.D.B.), U01HG007598 (M.O., B.E.S.), U01MH104393 (A.P.F.), extension H002371 to 5U41HG002371 (W.J.K) as well as other funding sources: R01MH106842 (T.L., P.M., E.F., P.J.H.), R01HL142028 (T.L., Si.Ka., P.J.H.), R01GM122924 (T.L., S.E.C.), R01MH107666 (H.K.I.), P30DK020595 (H.K.I.), UM1HG008901 (T.L.), R01GM124486 (T.L.), R01HG010067 (Y.Pa.), R01HG002585 (G.Wa., M.St.), Gordon and Betty Moore Foundation GBMF 4559 (G.Wa., M.St.), 1K99HG009916-01 (S.E.C.), R01HG006855 (Se.Ka., R.E.H.), BIO2015-70777-P, Ministerio de Economia y Competitividad and FEDER funds (M.M-A., V.W., R.G., D.G-M.), la Caixa Foundation ID 100010434 under agreement LCF/BQ/SO15/52260001 (D.G-M.), NIH CTSA grant UL1TR002550-01 (P.M.), Marie-Skłodowska Curie fellowship H2020 Grant 706636 (S.K-H.), R35HG010718 (E.R.G.), FPU15/03635, Ministerio de Educación, Cultura y Deporte (M.M-A.), R01MH109905, 1R01HG010480 (A.Ba.), Searle Scholar Program (A.Ba.), R01HG008150 (S.B.M.), 5T32HG000044-22, NHGRI Institutional Training Grant in Genome Science (N.R.G.), EU IMI program (UE7-DIRECT-115317-1) (E.T.D., A.V.), FNS funded project RNA1 (31003A_149984) (E.T.D., A.V.), DK110919 (F.H.), F32HG009987 (F.H.), Massachusetts Lions Eye Research Fund Grant (A.R.H.).

Competing interests

F.A. is an inventor on a patent application related to TensorQTL; S.E.C. is a co-founder, chief technology officer and stock owner at Variant Bio; E.R.G. is on the Editorial Board of Circulation Research, and does consulting for the City of Hope / Beckman Research Institut; E.T.D. is chairman and member of the board of Hybridstat LTD.; B.E.E. is on the scientific advisory boards of Celsius Therapeutics and Freenome; G.G. receives research funds from IBM and Pharmacyclics, and is an inventor on patent applications related to MuTect, ABSOLUTE, MutSig, MSMuTect, MSMutSig, POLYSOLVER and TensorQTL. G.G. is a founder, consultant and holds privately held equity in Scorpion Therapeutics; S.B.M. is on the scientific advisory board of MyOme; D.G.M. is a co-founder with equity in Goldfinch Bio, and has received research support from AbbVie, Astellas, Biogen, BioMarin, Eisai, Merck, Pfizer, and Sanofi-Genzyme; H.K.I. has received speaker honoraria from GSK and AbbVie.; T.L. is a scientific advisory board member of Variant Bio with equity and Goldfinch Bio. P.F. is member of the scientific advisory boards of Fabric Genomics, Inc., and Eagle Genomes, Ltd. P.G.F. is a partner of Bioinf2Bio.
